# Alternative Path Communication in Wide-Scale Cluster-Tree Wireless Sensor Networks Using Inactive Periods

**DOI:** 10.3390/s17051049

**Published:** 2017-05-06

**Authors:** Erico Leão, Carlos Montez, Ricardo Moraes, Paulo Portugal, Francisco Vasques

**Affiliations:** 1 Department of Computing, Federal University of Piauí, 64049-550 Teresina, Brazil; 2 INEGI/INESC-TEC, Faculty of Engineering, University of Porto, 4200-465 Porto, Portugal; pportugal@fe.up.pt (P.P.); vasques@fe.up.pt (F.V.); 3 Automation and Systems Department, Federal University of Santa Catarina, 88040-900 Florianópolis, Brazil; carlos.montez@ufsc.br; 4 Department of Computing, Federal University of Santa Catarina, 88905-120 Araranguá, Brazil; ricardo.moraes@ufsc.br

**Keywords:** IEEE 802.15.4, ZigBee, Wireless Sensor Networks, cluster-tree, ARounD, wide-scale, message streams

## Abstract

The IEEE 802.15.4/ZigBee cluster-tree topology is a suitable technology to deploy wide-scale Wireless Sensor Networks (WSNs). These networks are usually designed to support convergecast traffic, where all communication paths go through the PAN (Personal Area Network) coordinator. Nevertheless, peer-to-peer communication relationships may be also required for different types of WSN applications. That is the typical case of sensor and actuator networks, where local control loops must be closed using a reduced number of communication hops. The use of communication schemes optimised just for the support of convergecast traffic may result in higher network congestion and in a potentially higher number of communication hops. Within this context, this paper proposes an *Alternative-Route Definition* (ARounD) communication scheme for WSNs. The underlying idea of ARounD is to setup alternative communication paths between specific source and destination nodes, avoiding congested cluster-tree paths. These alternative paths consider shorter inter-cluster paths, using a set of intermediate nodes to relay messages during their inactive periods in the cluster-tree network. Simulation results show that the ARounD communication scheme can significantly decrease the end-to-end communication delay, when compared to the use of standard cluster-tree communication schemes. Moreover, the ARounD communication scheme is able to reduce the network congestion around the PAN coordinator, enabling the reduction of the number of message drops due to queue overflows in the cluster-tree network.

## 1. Introduction

Wireless Sensor Networks (WSNs) are special ad hoc networks composed of a large number of low-cost, low-power, and low-rate wireless devices (i.e., sensor nodes) with capability for sensing and, occasionally, actuating upon the environment where they are deployed. WSNs can be used to support multiple types of applications, such as: health, structural and environmental monitoring, home automation, vehicular systems, military applications, and many others [1,2,3]. In addition, due to their specific characteristics such as: flexibility, mobility, autonomous and collaborative operation, WSNs can be deployed in hazardous or hostile environments, where the human presence or the use of wired systems are unsuitable [4].

Recent advances in Micro-Electro-Mechanical systems (MEMs), microprocessors and low power radio technologies [2,3] enable the availability of sensor devices increasingly robust, with more storage and processing capabilities, and at a lower price. In this context, the implementation of WSN wide-scale applications with energy-efficient and time-sensitive requirements have become an attractive research topic.

The IEEE 802.15.4 standard [5] and the ZigBee specification [6] define a set of networking topologies commonly used in WSNs. The IEEE 802.15.4 defines PHYsical (PHY) and Medium Access Control (MAC) sublayers for Low-Rate Wireless Personal Area Network (LR-WPAN), focusing on short-range operation, low-data rate, energy-efficiency and low-cost implementations. The IEEE 802.15.4 standard basically defines two types of devices: a Full-Function Device (FFD) and a Reduced-Function Device (RFD). A FFD is capable of serving as PAN coordinator, cluster coordinator, or simple device, while a RFD is only capable of serving as simple device. Besides that, while FFD nodes can perform routing and management activities, RFD nodes are just able to communicate directly with their coordinator nodes. A Wireless Personal Area Network (WPAN) is composed of multiple FFD and RFD devices, with a unique FFD acting as PAN coordinator.

IEEE 802.15.4 defines two types of topologies: star and peer-to-peer. In a star topology, all sensor nodes directly communicate with the PAN coordinator. Thus, the network range is limited by the transmission range of its nodes, which is an impairment for wide-scale deployments. On the other hand, peer-to-peer topologies allow the implementation of more complex network formations, such as mesh and cluster-tree.

Mesh networking topologies use a decentralised communication paradigm, allowing multihop routing from any node to any neighbour node. Mesh topologies allow network flexibility and provide high routing redundancy and good scalability [7,8]. However, due to the lack of synchronisation mechanisms of traditional mesh networks, this topology type commonly does not allow nodes to enter into low power mode to save energy [9,10], which significantly reduces the network lifetime. Moreover, it introduces additional complexity to ensure end-to-end connectivity [7]. These characteristics are undesirable for typical WSN-based monitoring applications, where power consumption and lower complexity are crucial issues. The IEEE 802.15.4e-2012 standard [11] was released to enhance the real-time properties of IEEE 802.15.4 MAC protocol and to increase the network scalability by using the DSME (Deterministic and Synchronous Multi-channel Extension) protocol, that provides synchronisation mechanisms for mesh topologies. The DSME protocol was incorporated into the newest version of the IEEE 802.15.4-2015 standard. This extension is out of the scope of this work, which is focused on the single-channel version of the IEEE 802.15.4 protocol used by the majority of WSN deployments.

The cluster-tree hierarchical topology enables setting-up wide-scale deployments through a mesh of multiple neighbouring clusters. Therefore, cluster-tree topologies are preferable for setting-up wide-scale monitoring applications. The IEEE 802.15.4 standard does not describe by itself the required mechanisms to deal with cluster-tree topologies. It has been complemented by the ZigBee specification [6] that defines both network and application layers. In the network layer, ZigBee defines a set of rules for network formation, addressing and routing, providing mechanisms to build cluster-tree networks. These mechanisms employ a hierarchical addressing scheme and use tree-based routing algorithms. Cluster-tree topologies have the benefits of being beacon-oriented, which allows synchronisation, collision-free time slots, and duty-cycle operation. However, they still suffer from some relevant limitations in what concerns real-time aspects (latency and communication delays) and energy-efficiency [12,13], beacon frame collisions [7,14,15,16,17] and congestion control [18]. Additionally, as all cluster-tree paths go through the PAN coordinator, communications are prone to higher delays whenever the coordinator is not the final destination of the message streams [19].

### 1.1. Application Domains

**Figure 1:**
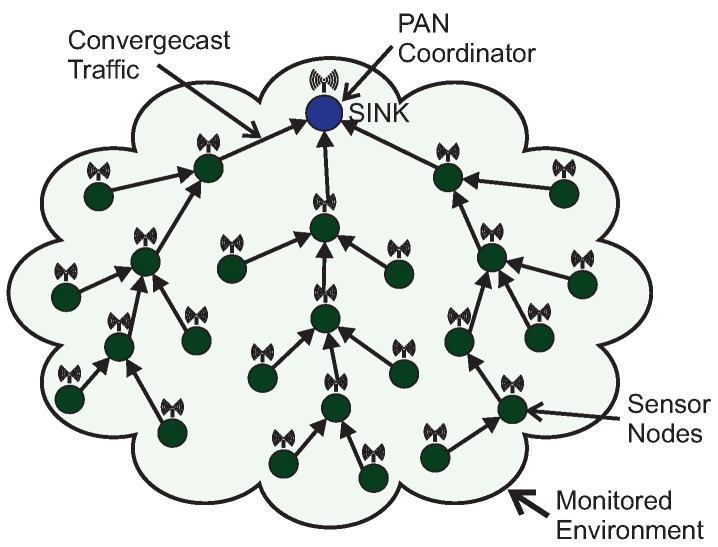
Typical convergecast communication traffic in a Wireless Sensor Network.

Operating mechanisms in Wireless Sensor Networks are usually application-dependent, in what concerns: node deployment, network topology, communication traffic patterns and communication protocols [20,21,22,23,24,25]. Regarding WSN traffic patterns, convergecast traffic is the most common traffic pattern in WSNs [26,27,28]. Convergecast traffic corresponds to typical sensing traffic (upstream) being periodically generated by sensor nodes and forward towards a sink node (usually at the PAN coordinator). Figure 1 illustrates a typical monitoring cluster-tree WSN, where static sensor nodes continuously transmit convergecast traffic.

**Figure 2:**
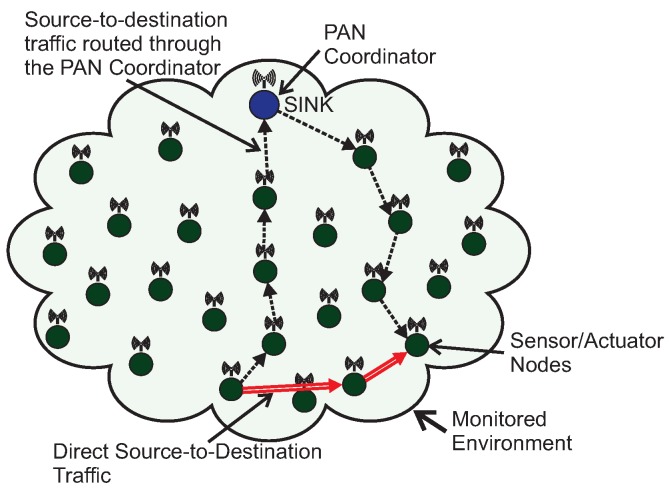
Source-to-destination traffic in a Wireless Sensor Network.

Other applications may require multiple destination nodes for message streams, i.e., WSNs can be designed with one or more sink nodes, depending on the application requirements [21]. This behaviour is especially observed whenever sensor nodes are deployed with actuating capabilities. Remarkable examples of multi-sink scenarios are local irrigation management systems in precision agriculture or smart control for shading systems in building automation [1,29,30,31], where actuating commands are generated according to locally sensed variables. Other applications are related to collaboration purposes, where sensor nodes can exchange messages with neighbour nodes to take local decisions, for instance detection of specific events in a given monitored environment [28,32]. In this type of sensing/actuating (or collaborative) applications, the network can be required to implement local control loops, based on local variables, without the intervention of a global controller (located at the sink node). Therefore, it would be of major relevance to have WSNs where any pair of nodes can be source/destination of message streams [33]. Figure 2 illustrates this type of source-to-destination communication pattern.

A major limitation when using traditional cluster-tree networks to support source-to-destination traffic is that data communication must follow the tree routing paths (dashed arrows in Figure 2). Thus, if source and destination nodes are not located in the same cluster, the message stream will be required to traverse multiple clusters towards the PAN coordinator (or, at least, a common parent cluster-head) and then down until reaching the destination node. This type of routing scheme generates higher end-to-end communication delays and potentially higher network congestion of the cluster-tree backbone nodes.

Within this context, the communication scheme proposed in this paper uses available communication resources to set alternative communication paths, enabling the support of direct source-to-destination message streams (double arrows in Figure 2). Although being widely studied in MANET (Mobile Ad hoc Networks) literature [33,34,35], there are two fundamental differences in what concerns the application domains for these alternative routing paths: ARounD considers WSN clusters where sensor nodes are most part of the time in sleeping mode, in order to save energy; and there are no mobile nodes in the communication environment. Instead, there is a set of static WSN nodes that may be used during their idle periods to support the proposed alternative routing paths.

### 1.2. Objectives and Contributions of This Paper

In this paper, we propose the *Alternative-Route Definition* (ARounD) communication scheme. This scheme is able to support source-to-destination message streams in cluster-tree WSNs, without relaying messages through the PAN coordinator (root node). The proposed communication scheme defines alternative communication paths, which are set without interfering with the pre-defined cluster-tree communication paths. The underlying idea is to search for new inter-cluster mesh paths compatible with the previously defined beacon scheduling, using available border nodes of the clusters during their inactive periods. These alternative paths typically go through less congested areas of the network, which may result in smaller end-to-end communication delays.

To the best of our knowledge, this is the first study that proposes the use of WSN border nodes during their inactive periods to provide shorter communication paths, avoiding interferences with the previously scheduled cluster-tree communication. The importance of the proposed ARounD scheme can be highlighted by the emergence of applications with source-to-destination message streams, such as those found in WSNs with actuating capabilities.

### 1.3. Organisation of This Paper

This paper is organised as follows: Section 2 provides the required background for the proposed ARounD scheme. Section 2.1 presents an overview of the IEEE 802.15.4/ZigBee cluster-tree topology and Section 2.2 presents the state-of-the-art on routing protocols for wireless sensor networks, highlighting applications for wide-scale wireless sensor networks, cluster-tree topologies and beacon frame scheduling. Section 3 presents the proposed ARounD communication scheme, highlighting its main algorithms and protocol mechanisms. Section 4 presents a simulation assessment of the proposed communication scheme. Finally, in Section 5, some conclusions and future considerations are presented.

## 2. Background

### 2.1. IEEE 802.15.4/ZigBee Cluster-Tree Topologies

A cluster-tree topology is a special case of a peer-to-peer network. In a cluster-tree topology, nodes/devices are grouped in clusters, coordinated by a FFD node called Cluster-Head (CH). The CH provides synchronisation mechanisms for its associated nodes and centralises all intra-cluster communication. The cluster-tree network formation is initiated by the PAN coordinator, which acts as coordinator for the network, being responsible for all network management activities.

**Figure 3:**
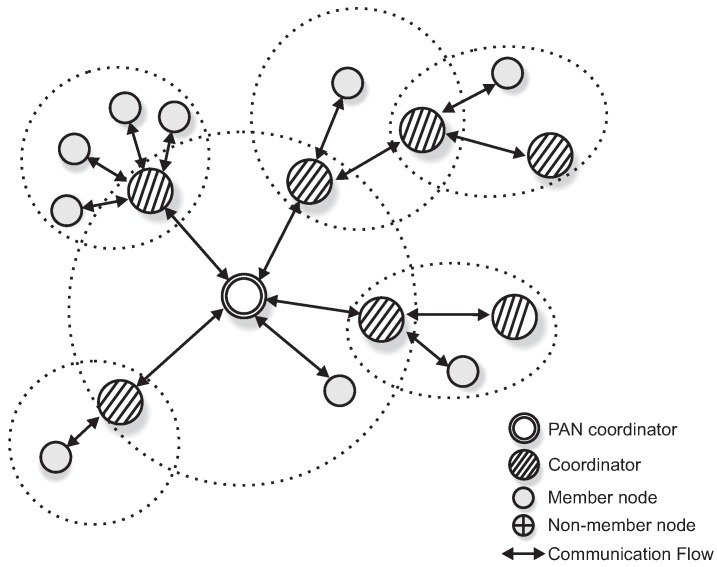
Cluster-tree networking topology.

According to the IEEE 802.15.4 standard, the simplest case of a cluster-tree network is a single cluster (coordinated by the PAN coordinator). New nodes may be allowed to create their own clusters, increasing the coverage of the network. Within this context, several neighbouring clusters can be used to build wide-scale cluster-tree networks, where the coordinators are connected by parent-child relationships, forming a multicluster hierarchical network structure. Figure 3 shows an example of an IEEE 802.15.4 cluster-tree network.

Although considering cluster-tree networks, the IEEE 802.15.4 standard does not discuss the required network formation mechanisms. The ZigBee specification provides these mechanisms, defining a hierarchical addressing scheme and the associated tree routing algorithms, where network addresses are assigned based on address blocks. Thus, each cluster-head has its own block of sequential addresses, which are assigned to its child nodes. Based on this hierarchical addressing, ZigBee also provides a deterministic tree routing scheme. In this scheme, routing is based on the destination address. In case the destination address is a descendant node, the packet is forward to the corresponding child node; otherwise, the packet is forward to the parent node.

**Figure 4:**
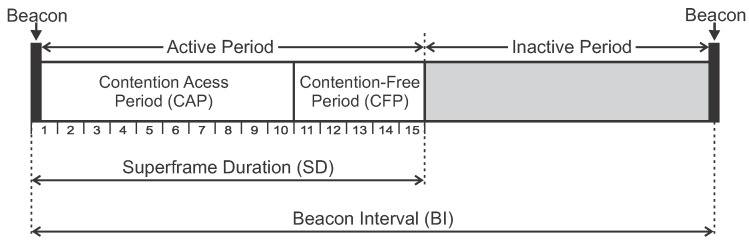
IEEE 802.15.4 Superframe structure.

In cluster-tree topologies, the network operates in a beacon-enabled mode. In this mode, communication exchanges are organised according to a structure called Superframe. A superframe is bounded by beacon frames periodically transmitted by the coordinators (cluster-heads). These beacon frames synchronise the associated nodes, identify the PAN and describe the superframe structure. Figure 4 illustrates the superframe structure.

The superframe is described by the *macBeaconOrder* (BO) and *macSuperframeOrder* (SO) parameters, where Beacon Interval (BI) and the Superframe Duration (SD) are defined as follows:

(1) $$\begin{array}{c} BI=aBaseSuperframeDuration\times 2^{BO}\left(symbols\right) \end{array}$$

(2) $$\begin{array}{c} SD=aBaseSuperframeDuration\times 2^{SO}\left(symbols\right), \end{array}$$

with 0≤SO≤BO≤14.

BI defines the interval at which the coordinator periodically transmits beacon frames. In turn, the SD parameter defines the length of the active portion of the superframe. The aBaseSuperframeDuration parameter defines the minimum duration of the superframe when SO is 0. The IEEE 802.15.4 standard defines this parameter with 960 symbols duration, which corresponds to 15.36 ms (assuming a network with bit rate of 250 kbps, frequency band of 2.4 GHz, and one symbol as 4 bits).

Each superframe is divided in two parts: the active and inactive periods. During the inactive period, the coordinator and associated nodes can enter in low power mode to save energy (sleep mode). During the active period, nodes can communicate with the coordinator. The active part (communication period) comprises two periods: Contention Access Period (CAP) and Contention-Free Period (CFP).

In the CAP period, whenever a device wishes to communicate, it contends with other devices using a slotted Carrier Sense Multiple Access with Collision Avoidance (CSMA-CA) procedure to access the channel. The CFP is defined for applications that require low latency or specific bandwidth. In the CFP period, the coordinator allocates Guaranteed Time Slots (GTS) for specific nodes. In these slots, nodes can transmit data without contending for the channel access. The coordinator can allocate up to seven GTSs, and a GTS is allowed to occupy more than one slot period [5].

In the cluster-tree topology, a coordinator node (excluded the PAN coordinator) must keep the synchronisation between its own active period (acting as coordinator node) and the parent’s active period (acting as child node). Also, for each cluster, low duty cycles can be activated to save energy, setting a value of SO smaller than BO.

In cluster-tree networks, sending beacon frames without any special care on timing issues may result in collisions among beacons from neighbour clusters [7]. Thus, it is necessary to implement inter-cluster synchronisation mechanisms to avoid this problem. Two types of beacon collisions are possible: direct or indirect. In a direct beacon collision, two or more coordinators (in the transmission range of each other) transmit their beacon frames at the same time. In the indirect beacon frame collision, two or more coordinators are hidden-nodes from each other and send messages to an overlapped node [7].

This paper is focused on cluster-tree networks defined by the ZigBee specification [6], due to some specific features, such as: suitability to deploy wide-scale networks, energy-efficiency and time-sensitive message guarantees.

### 2.2. Related Work

In recent years, due to the high demand of applications supported by wireless sensor networks, there have been a large number of works addressing cluster-tree WSNs. Multiple issues are being addressed, such as: network formation schemes, routing protocols, cross-layer approaches, real-time requirements, reliability and availability issues, energy-efficiency, scalability, congestion control and beacon frames scheduling. Each of these issues have their own challenges and special considerations. Additionally, with the increased demand for wide-scale applications that need to operate with time-sensitive data and must be energy-efficient, the design of wide-scale WSNs is becoming more challenging due to the large number of constraints that need to be simultaneously addressed.

The state-of-the-art indicates that the use of hierarchical clustering protocols has some advantages over flat protocols for wide-scale applications with respect to scalability [36,37,38], energy-efficiency [7,14,36,38,39] and time-sensitive data operation [7,14,15,38,39,40]. Therefore, several works have been published in the literature, encompassing clustering for WSNs, with different concerns. As an example, there are several surveys that summarise popular clustering protocols and analyse the strengths and weaknesses of available routing protocols based on different metrics [41,42,43,44,45,46]. For example, LEACH (Low Energy Adaptive Clustering Hierarchy) protocol [47] is one of the most cited hierarchical protocols in the literature, which has motivated the design of several other protocols. In LEACH, sensor nodes are grouped in clusters for sending data to their cluster-heads, which aggregate data and send it to the base station. In order to balance the energy consumption of sensor nodes, this protocol randomly selects a set of different cluster-heads during each round. Also, Kumar et al. [36] proposed an Energy Efficient Heterogeneous Clustered Scheme (EEHC) based on the LEACH protocol, considering the heterogeneity of network resources. However, as it uses single-hop routing, the cluster-heads send directly data to the base station, which leads to an increase of energy consumption. Thus, this type of communication may be impracticable for wide-scale environments.

Additionally, several cross-layer approaches have been proposed to deal with energy-efficient communication in Wireless Sensor Networks [48,49,50,51,52]. Typically, cross-layer approaches blend common protocol layer functionalities into a cross-layer module, addressing simultaneously congestion control, routing and medium access control issues to enhance the efficiency of the traditional layered protocol architectures.

As the communication scheme proposed in this paper addresses setting-up new communication paths in wide-scale cluster-tree networks, this state-of-the-art study does not consider the use of cross-layer approaches, as the use of this type of architectures would not be compatible with the use of standard IEEE 802.15.4/ZigBee cluster-tree networks. Instead, it is focused on relevant approaches that can be implemented upon cluster-tree networks, regarding cluster-tree formation, its synchronisation, congestion control and data communication support in wide-scale cluster-tree networks.

Within this context, Zhu et al. [53] proposed a Tree-Cluster-Based Data-Gathering Algorithm (TCBDGA) using a mobile sink to improve the non-uniform energy consumption of WSNs. The basic idea of this algorithm is to build a cluster-tree network considering specific characteristics of the nodes, such as their residual energy, distance to base station and number of neighbours. After this, the network is decomposed in several sub-trees and each sub-tree has its own set of collecting nodes. The mobile sink is responsible to collect data from nodes. This algorithm is a location-aware based algorithm, which implies more energy consumption and complexity. The communication approach proposed in this paper considers a static topology (no mobile nodes), where the information is gathered through message exchanges.

Choi and Lee [13] implemented a multihop GTS mechanism for IEEE 802.15.4 beacon-enabled networks. The aim of this work is to allocate GTS slots from the requesting nodes to the sink node along the cluster-to-cluster path. In turn, Felske et al. [19] proposed the GLHOVE approach for cluster-tree networks to maximise the number of received messages in the base station, prioritizing specific clusters of the network. GLHOVE comprises a distributed algorithm that guarantees a balanced message delivery per cluster and, at the same time, avoiding congestion. However, none of these approaches consider any message prioritisation mechanism.

Khatiri et al. [54] and Kim et al. [55] proposed two different approaches to decrease the number of hops to reach the sink node using the neighbouring table defined in the ZigBee standard. The purpose is to reduce the routing costs, improving the energy consumption, network lifetime and end-to-end delays. In [54], each node keeps a neighbouring table with relevant information about the neighbouring nodes, such as: depth, link quality and device type. Thus, the algorithm defines the shortest path using three criteria: minimum hop count, minimum congestion and maximum link quality. Each criterion has its weight and the next hop (neighbour node) is selected based on a minimum cost function. In [55], the algorithm inspects the neighbouring table in order to find a node with a shortest tree path until the destination node and selects it as next hop. Although these algorithms define shortest paths until the sink node, they do not consider any information about network scheduling (top-down or bottom-up), nor about how data communication is performed.

Misic [56] presented an approach using border nodes (slave-slave bridge) to interconnect neighbouring clusters. The slave-slave bridge nodes listen to the beacons generated by source and sink clusters. During the source cluster active period, the bridge node can receive data from the coordinator, and during the sink cluster active period, the bridge node delivers its data to the sink coordinator. Considering this approach, the cluster-tree path is not used. Although using bridge nodes to avoid the cluster-tree path, data is transmitted during the clusters’ active period, and therefore, end-to-end delays are still dependent of the cluster scheduling.

Also, Huang et al. [57] proposed an adoptive-parent-based framework for ZigBee networks, to increase the bandwidth between the source and sink nodes. For this, a specific cluster-head can request bandwidth from neighbour cluster-heads (adoptive parents) during a given time period. However, even using additional paths to transmit messages between source and sink nodes, these additional paths are cluster-tree paths and therefore the problem of a higher energy consumption of the cluster-heads still remains. The communication scheme proposed in this paper defines new alternative paths for message streams, instead of cluster-tree paths, which can improve end-to-end communication delays and reduce network congestion.

Regarding beacon frame scheduling, some relevant works have also been presented in [7,14,15,16,39,58]. The key idea of these works is to schedule the clusters’ active period, considering the main constraints imposed by the message streams supported by the cluster-tree network.

Toscano and Lo Bello [15] and Abdeddaim et al. [58] follow a multichannel approach to avoid overlapping cluster collisions, while maintaining the cluster connectivity. In [15], the proposed approach schedules adjacent clusters in alternate timeslices, using different channels to avoid cross-channel interferences. Thus, while a coordinator schedules its own superframe, its adjacent clusters can not schedule their owns. Therefore, collisions are avoided between neighbour clusters. Following this approach, a specific cluster is able to receive the parent’s superframe in a given timeslice and schedule its superframe in an adjacent timeslice. The clusters are scheduled within a major cycle, which is cyclically repeated. In order to avoid inter-cluster collisions, a set composed of the PAN coordinator and all the clusters that can be reached in an even number of hops are scheduled in a given timeslice; then, all other clusters are scheduled in alternate timeslices. Abdeddaim et al. [58] proposed an IEEE 802.15.4-based cluster-tree formation protocol, named Multi-Channel Cluster Tree (MCCT), which uses multiplexed transmissions across different channels, in order to avoid beacon collisions. In addition, it uses a shared control channel for the cluster-tree construction and maintenance operations. Thus, a node just scans the control channel to request an association. The joining node defines its parent cluster-head based on the number of associated children. The cluster-head with lower number of children is selected. Thus, the association process occurs during the active period, using the channel of the specific cluster-head. These works use multi-channel approaches in order to avoid beacon collisions, which add overheads related to the channel’s maintenance and control.

Koubaa et al. [7] proposed the Time Division Beacon Frame Scheduling (TDBS) approach, which defines a Superframe Duration Scheduling (SDS) algorithm for cluster-tree networks. In this approach, superframe duration and beacon frames of a given cluster are scheduled in the inactive periods of its neighbour clusters to avoid inter-cluster interferences. The schedulability condition of SDS is both necessary and sufficient, and is obtained considering the duty cycle information of nodes [15]. The TDBS approach defines minor and major cycles to schedule a cluster set with different superframe durations and beacon intervals. All clusters are organised within the defined major cycle, based on the Least Common Multiple (LCM) of the beacon periodicities for all clusters. The major cycle is divided in minor cycles, which are used to sequentially fit all clusters. In this approach, clusters are organised in an increasing order of beacon periodicities. To break ties, these clusters are organised in a decreasing order of superframe durations. Clusters are sequentially organised within the minor cycles, until reaching the end of the major cycle. TDBS defines the start time for all clusters in a collision-free scheduling scheme. However, it does not consider any message stream prioritisation and therefore may not be adequate to support time-sensitive applications.

Hanzalek and Jurcík [14] present a Time-Division Cluster Scheduling (TDCS) mechanism to avoid inter-cluster collisions and to meet all end-to-end deadlines of time-bounded message streams. This mechanism employs a pure time-division scheduling approach, avoiding the inter-cluster collision problem. Besides, it aims to define the maximum TDCS period (major cycle) in order to minimise the energy consumption of the nodes. This is a challenging task because it is necessary to consider the message stream requirements, meeting all their end-to-end deadlines. To solve this problem, the authors formulate the TCDS approach as a cyclic extension of the Resource Constrained Project Scheduling with Temporal Constraints (RCPS/TC), which defines a feasible schedule considering the temporal and resource constraints of a set of tasks. After modelling this problem, they use an integer linear programming algorithm to solve the scheduling problem.

The main weakness of TDBS and TDCS approaches is the use of an off-line scheduling approach, that assumes static network conditions. Thus, Severino et al. [39] presented an interesting alternative to modify the scheduling at run-time, in order to provide QoS (Quality of Service) to message streams. Basically, they proposed the use of two different techniques: Dynamic Cluster Re-ordering (DCR) and Dynamic Bandwidth Re-allocation (DBR). The DCR technique is used to re-order at run-time the cluster scheduling. The clusters involved in the data streams are re-ordered, in order to minimise the traffic latency. This re-scheduling is performed based on the priority of the data streams, in order to decrease end-to-end communication delays. In turn, the DBR technique is used to change the superframe duration of the clusters, increasing the bandwidth of the clusters involved in the message stream. This technique uses the free space (not used to allocate clusters) and distributes it among the involved clusters. If there is no available free space, it tries to reduce the bandwidth of the non-involved clusters, in order to re-distribute it among the involved clusters. Unfortunately, this communication approach only considers the use of cluster-tree paths.

While TDBS and TDCS approaches just address upstream communication traffic (convergecast pattern), Yeh and Pan [16] proposed an efficient beacon scheduling approach to support low-latency upstream and downstream traffic. The authors formulate the Low-latency Two-way Beacon Scheduling (LTBS) problem for ZigBee networks. In this problem, nodes try to get slots for upstream and downstream traffic, while avoiding interferences from other clusters. LTBS modifies the IEEE 802.15.4 original superframe structure, in order to allow each cluster-head to broadcast two beacons: one for upstream direction and another for downstream direction. The authors propose two algorithms (centralised and distributed) to assign interference-free upstream and downstream slots to reduce the network latency. Basically, the difference between centralised and distributed approaches is the sequencing order. The centralised algorithm assigns slots using a bottom-up approach. On the other hand, the distributed algorithm assigns slots using a top-down approach. This proposal minimises the network latency without considering any message stream prioritisation.

Although improving the performance of cluster-tree networks, the efficiency of the proposed communication schemes is upper-bounded by the cluster-tree routing scheme and its inherent problems, such as: network congestion near the PAN coordinator [19], poor bandwidth allocation to the different clusters, and higher energy consumption of crucial nodes.

## 3. Alternative Paths for Message Streams in Cluster-Tree Wireless Sensor Networks

In this paper, we propose a new communication scheme for cluster-tree wireless sensor networks, called *Alternative-Route Definition* (ARounD). The underlying idea is to define alternative communication paths avoiding congested areas of the network. The ARounD communication paths avoid interferences with the previously defined cluster-tree paths, by means of a careful selection of the timing instants when messages their are transferred.

The proposed ARounD communication scheme is intended for wide-scale applications requiring the support of source-to-destination message streams. It improves several performance metrics of wide-scale cluster-tree networks, considering that: (1) by defining alternative communication paths, it decreases the network congestion around the PAN coordinator; (2) by selecting shorter inter-cluster paths, it can potentially decrease end-to-end communication delays of message streams; and (3) it is able to guarantee a higher level of Quality-of-Service (QoS) for source-to-destination message streams, as the involved nodes will access the medium without contention.

In order to establish the new source-to-destination paths, the ARounD communication scheme adds new functionalities for specific cluster-tree nodes and defines two main algorithms: *Alternative-Path Definition* (ARounD-Def) and the *Alternative-Path Activation* (ARounD-Act). In the next subsections, we describe both the network model and the ARounD communication algorithms.

### 3.1. Network Model

Assume a set of sensor nodes randomly deployed in a wide-scale environment. These nodes are organised in clusters according to the IEEE 802.15.4/ZigBee cluster-tree topology [5,6]. It is worth mentioning that whenever there are link breakages or modifications of the network topology, there will be the need to rebuild the network. This reorganisation may be done following a strategy similar to the ones proposed by [7,59]. For the sake of simplicity, we assume a static set of clusters and we also assume that the network topology does not change along the network lifetime. The network is composed of *N* coordinator nodes (including the PAN coordinator), that act as Cluster-Heads (*CHs*) of their clusters and periodically send beacon frames to synchronise their child nodes, such that:

(3) $$CH_{i}=\left(SD_{i},BI_{i}\right),\;1\leq i\leq N,$$

where *i* is a unique identifier for the specific cluster, $SD_{i}$ corresponds to the *Superframe Duration* and $BI_{i}$ is the *Beacon Interval* of the cluster-head $CH_{i}$. We assume that values for BO and SO are defined according to the load supported by each of the branches of the cluster-tree, considering a superframe duration allocation approach, as the one proposed by [59]. We also assume that the cluster’s active periods were previously scheduled, considering a classical time division superframe scheduling approach, as the one proposed by [7]. Therefore, there are no beacon frame collisions caused by overlapping clusters. Importantly, for each node is assigned a hierarchical global address and all nodes operate over the same communication channel.

**Figure 5:**
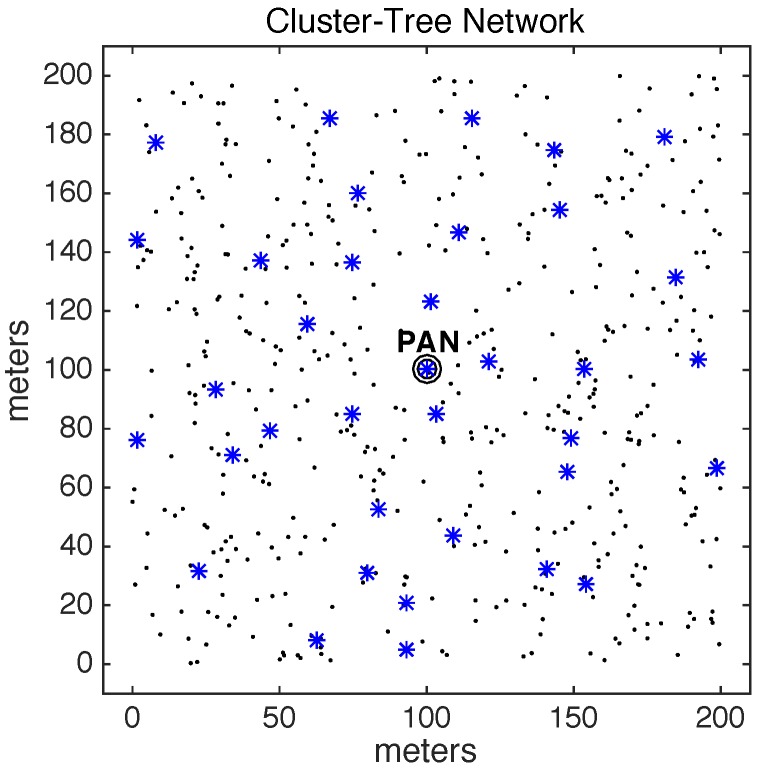
Cluster-tree network obtained from simulations.

Figure 5 illustrates an example of this random cluster-tree network with 200 nodes. The PAN coordinator is located in the centre of the wide-scale communication environment. The dot marks represent leaf nodes and the asterisk marks represent cluster-head nodes.

**Figure 6:**
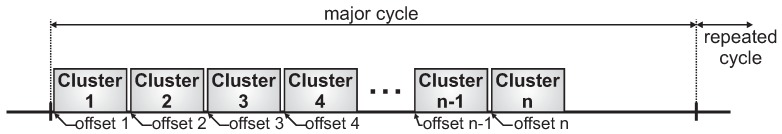
Cluster’s active period scheduling along the time.

Figure 6 shows a typical time division superframe scheduling scheme. As it can be seen, the cluster’s active periods are organised along the time, in order to avoid interferences. Thus, during the active period of their clusters, sensor nodes can communicate with their coordinators (cluster-heads) and vice-versa. During the inactive periods, sensor nodes can sleep to save energy.

**Figure 7:**
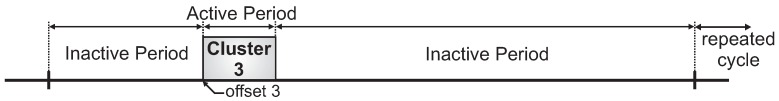
Duty cycle for the specific cluster 3, showing its active and inactive periods.

Figure 7 shows the duty cycle for cluster 3, considering a cluster-tree time division superframe scheduling. Note that the active period of cluster 3 starts at instant *offset* 3, where all of its sensor nodes wake up to sense the environment and to communicate. During the remaining inactive period, these nodes can sleep to save energy.

The proposed ARounD communication scheme uses two algorithms (Alternative-Path Definition and Alternative-Path Activation algorithms) to establish an alternative path for specific source-to-destination message streams. These algorithms use available communication resources during the inactive periods of the underlying cluster-tree network. Importantly, ARounD communication algorithms use the current network topology and information gathered during the cluster-tree formation process. Thus, a topology modification will require both a reconfiguration of the cluster-tree WSN and an update of network information, in order to keep the operation of the ARounD algorithms. This issue is inherent to any static approach used in cluster-tree WSNs. However, the assessment of the reconfiguration time that a cluster-tree network takes to reconfigure its topology is out of the scope of this work. In the next subsections, we detail these algorithms and the related ARounD structures.

### 3.2. Alternative-Path Definition Algorithm

The *Alternative-Path Definition* (ARounD-def) algorithm is performed by the PAN coordinator to find an alternative path for a given message stream. This way, after receiving a source-to-destination communication request, instead of considering the tree path, the PAN coordinator uses the available sensor nodes to define a *shorter inter-cluster path* between source and destination nodes. This shorter inter-cluster path is related to the smaller number of overlapping clusters that the message stream should traverse from the source to the destination. Currently, the *number of clusters* is being used, but other metrics could be also used to define the alternative path, e.g., *link quality*, *bandwidth* or *energy capacity*.

**Figure 8:**
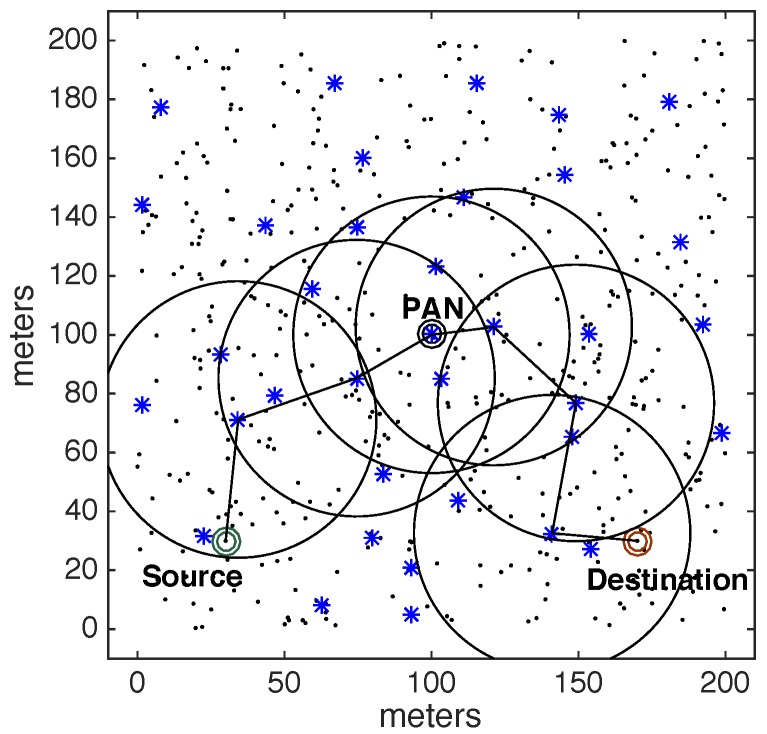
Standard cluster-tree path between the source and destination nodes.

Consider an example of a cluster-tree network, where the pair source-destination is located in specific positions in the communication environment as shown in Figure 8.

Figure 8 illustrates the cluster-tree path between the source and destination nodes. Along this path, each node must wait for the active period of their parent cluster, in order to communicate towards its cluster-head and send the data packets. According to this forwarding strategy, a message stream may face high end-to-end communication delays, which are highly dependent of the beacon scheduling strategy (favouring either bottom-up or top-down traffic, but not both) and of the network congestion behaviour near the PAN coordinator.

**Figure 9:**
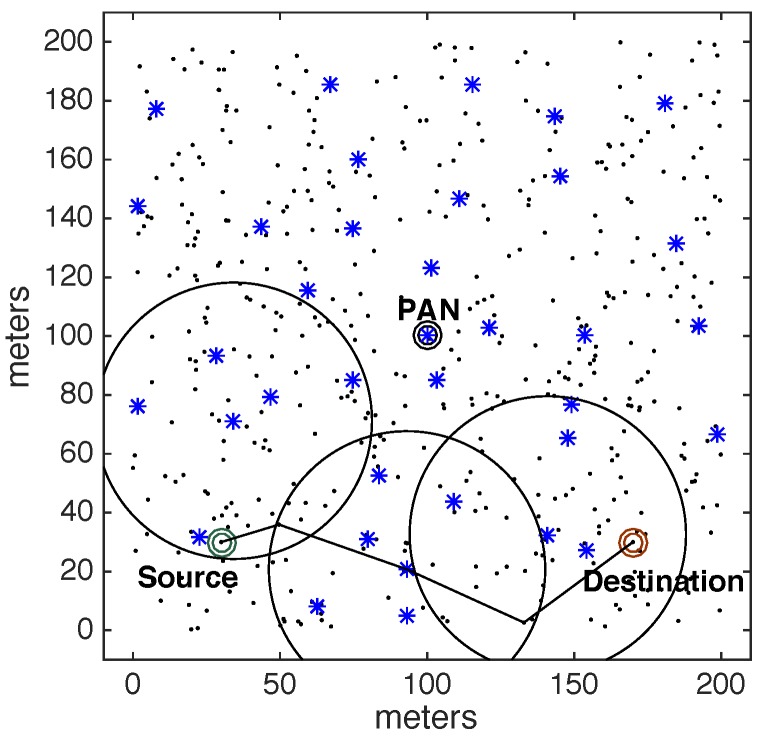
Alternative-Route Definition (ARounD) path between the source and destination nodes.

Instead, the ARounD communication scheme defines a different path, exploiting overlapping clusters, and building a shorter inter-cluster path between source and destination nodes. Figure 9 illustrates an ARounD path for the same example. Note that the defined path also traverses some clusters but does not necessarily use cluster-head nodes to relay data packets from source to destination nodes.

The ARounD communication scheme defines a set of special nodes: the *ARounD Repeater Nodes* (RP). This type of nodes are responsible for relaying data frames among overlapping clusters along the ARounD path, acting as border nodes between clusters. An RP node is a full function device that sits in the transmission range of two or more cluster-heads, independently of its functionality in the cluster-tree.

In order to represent repeater nodes and define the collision domain of the cluster-tree network, we use an adjacency list, which is a structure for the representation of the connectivity. Thus, considering a cluster-tree network with *N* cluster-heads, the ARounD scheme defines a set of adjacency lists, one adjacency list per cluster-head. For each $CH_{i}$ (1≤i≤N), it stores an array of the $CH_{j}$ (1≤j≤N) adjacent to it. Based on these adjacency lists, we define a list of edges $E=\left(RP_{i},RP_{j}\right)\in CH_{i}\;/\;1\leq i,j\leq N$, that are ordered based on their energy capacities. Importantly, both the adjacency lists and the RP nodes are defined during the formation phase of the cluster-tree network through message exchanges. The re-ordering of list *E* is done throughout the life of the WSN.

The first step of the ARounD-def algorithm (lines 2–5 in Algorithm 1) is to use adjacency lists to obtain an inter-cluster graph. Based on this graph, it has all possible inter-cluster paths from a source node to a destination node. With this, the PAN coordinator defines the *shortest inter-cluster path* between the pair of source and destination nodes, according to a shortest path algorithm (e.g., using the *Dijkstra’s* algorithm [60]), and considering just the set of edges with an energy level above a predefine threshold. In the current version of algorithm, we have used the remaining node energy level as input parameter for shortest path algorithm. However, other metrics can be used, such as the link quality level.

**Algorithm 1:**
*Alternative-Path Definition* (ARounD-def) Algorithm
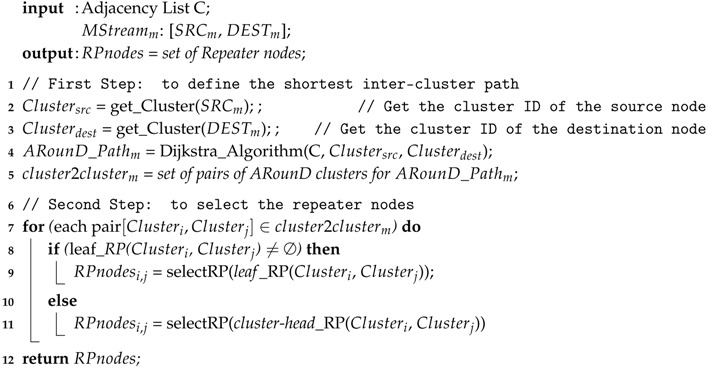


**Figure 10:**
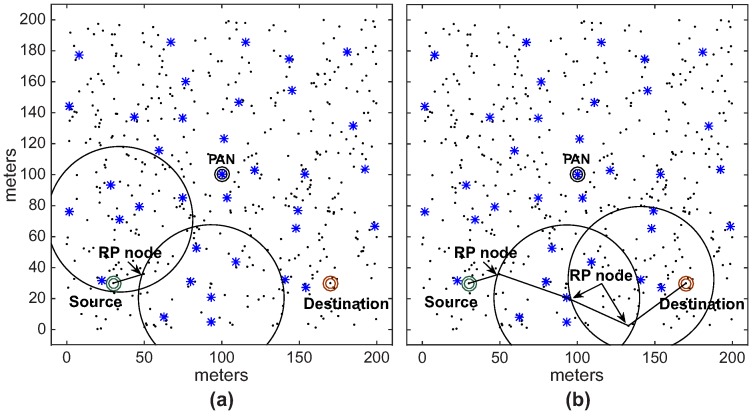
Applying the ARounD-def Algorithm: (**a**) Selecting repeater nodes for the first pair of clusters; (**b**) Selecting repeater nodes for the second pair of clusters.

After defining the shortest inter-cluster path (ARounD path), the next step is to select the repeater nodes that will relay data packets along this path. In order to structure and optimise this selection, we decompose this path (set of clusters) as a sequence of pairs of clusters (called *pairs of ARounD clusters*, lines 4 and 5 in Algorithm 1). For each pair of clusters, the selected repeater nodes are responsible for relaying data frames from the initial cluster to the next cluster. Figure 10 illustrates the selection of repeater nodes for the pairs of ARounD clusters. As it can be seen, the ARounD-def algorithm selects a specific repeater node for the first pair of clusters. Following the ARounD path, the algorithm selects repeater nodes for the second pair of clusters and, finally, the destination node is reached.

This selection process of repeater nodes uses an optimisation algorithm (lines 7–11 in Algorithm 1), in order to select the most adequate set of RP nodes. For this, ARounD keeps two lists of repeater nodes for each pair of clusters: list of *leaf* RPs, which contains RP nodes that act as leaf nodes in the cluster-tree network; and a list of *cluster-head* RPs, with RP nodes that act as cluster-heads. As long as we are dealing with full function devices, both type of RP nodes can act as repeater nodes during the ARounD communication. Importantly, in these two lists, RP nodes are ordered based on their energy capacities, being selected the node with more remaining energy. In order to select an RP node between a pair of clusters belonging to the ARounD path, the proposed scheme firstly inspects the list of *leaf* RPs, to save energy of cluster-heads that are responsible to keep the cluster-tree topology. If there are no available leaf RPs with a remaining energy level above the predefined threshold, the algorithm selects a cluster-head node to act as RP node in the ARounD path. The *selectRP* function (lines 9 and 11 in Algorithm 1) implements the mechanisms to select an RP node among the available RP nodes in each list. This function can be implemented using other selection criteria, such as: random approaches, link qualify of nodes or based on the physical position (location-aware protocols) [61,62].

After selecting the repeater nodes for each pair of ARounD clusters, the next step performed by the PAN coordinator is to define their activation times.

### 3.3. Alternative-Path Activation Algorithm

The *Alternative-Path Activation* (ARounD-act) algorithm defines the activation instants (schedule) for the selected RP nodes, which is also performed by the PAN coordinator. The activation instant of an RP node is a crucial issue. Due to the current cluster-tree communication, the ARounD communication must be adequately scheduled; otherwise, it can generate collisions and interferences with the previously scheduled cluster-tree communication. To prevent these problems, the ARounD communication scheme activates the RP nodes during their inactive periods and only when there is no activity in the neighbourhood.

We define the *ARounD Activity Period* as the period during which an RP node can be activated without interfering with the cluster-tree communication. Within this context, we define two types of ARounD activity periods, as follows:

- *Global ARounD Activity Period* (GAP): period during which there are no active clusters in the cluster-tree network;
- *Local ARounD Activity Period* (LAP): period during which there are no active clusters in the neighbourhood of the RP node.

The GAP period means that all nodes in the cluster-tree are inactive. Considering that the beacon interval (BI) must be large enough to ensure that all superframe durations ($SD_{i}$) can be scheduled and that there is also some available spare time during BI, the GAP period is defined based on the following protocol constraint:

(4) $$BI=GAP+\sum_{i=1}^{N}SD_{i},$$

Thus, during the GAP period, RP nodes can be activated without generating interferences with the underlying cluster-tree network.

In turn, the LAP period means that exists at least one activated cluster in the cluster-tree network, but that those clusters are not in the neighbourhood of the RP nodes. Therefore, a special care must be taken of when activating RP nodes, as these nodes may generate interferences with the cluster-tree network. Unlike the GAP period, which is the same for all RP nodes, the LAP period is dependent of the clusters in the neighbourhood of the RP nodes. For this reason, considering the ARounD path and the RP nodes, the ARounD-act algorithm avoids the active periods of the clusters and of their neighbours in the cluster-tree network. In this way, an RP node that becomes active to perform the ARounD communication does not generate interferences with the remaining clusters that are active in the cluster-tree network.

Thus, considering both the cluster-tree scheduling and the nodes that belong to the ARounD path (source, destination and RP nodes), the target of the ARounD-act algorithm is to define the activation instants for each of these nodes. For each ARounD node it will be defined a transmission time (ARounD Tx time) and a reception time (ARounD Rx Time), except for the source and destination nodes that will only have, respectively, a defined transmission and reception time.

When the ARounD-act algorithm (Algorithm 2) is launched, the PAN coordinator starts exploring the clusters’ active period scheduling (line 3 in Algorithm 2), in order to search the first *ARounD activity period* (GAP or LAP, but preferably GAP) that is larger or equal to the required period to allocate to the source node (as transmission node) and to the next RP node (as reception node) of the ARounD path. After, the algorithm searches for other *activity period* for this same RP node (now, as transmission node) and to the next RP node (as reception node). This procedure is recursively performed until reaching the destination node (lines 2–14 in Algorithm 2). Whenever the ARounD-act algorithm is not able to find any existing ARounD activity periods to allocate to all RP nodes (lines 13 and 14 in Algorithm 2), the algorithm fails and the next higher layer is notified. In case all RP nodes are successfully allocated, the ARounD-act algorithm defines a set of *ARounD_Offsets* (communication schedule), which correspond to the activation time for each RP node (lines 5–7 and 15 in Algorithm 2). As a consequence, each RP node will be awake at its respective *ARounD_Offset* time to perform the ARounD communication. This communication is then performed without contention, node-to-node, from the source node until reaching the destination node. Importantly, when activating a new alternative path, ARounD algorithms consider the current paths in order to avoid overlapping communication. Moreover, as each repeater node has its own activating times for each message stream, multiple ARounD paths can use common repeater nodes without generating interferences during the communication.

**Algorithm 2:**
*Alternative-Path Activation* (ARounD-act) Algorithm
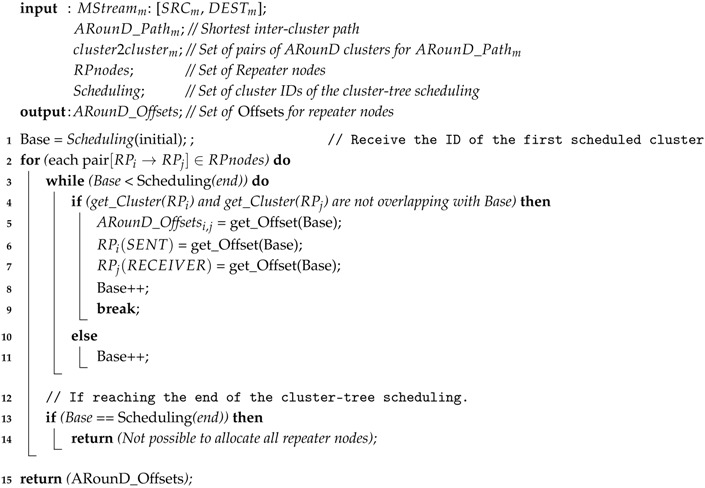


In order to reduce the configuration overhead, the ARounD path is automatically maintained during a time pre-defined during the configuration phase. After this period, the ARounD path will be terminated, unless a new request for message stream communication is performed.

### 3.4. ARounD Protocol Mechanisms

In this subsection, we present a set of protocol mechanisms that are required to establish the ARounD communication. After running the ARounD-def and ARounD-act algorithms, the PAN coordinator configures and synchronises all the involved nodes (*ARounD negotiation phase*). All these steps can be implemented without modifying the IEEE 802.15.4 standard. In fact, all ARounD protocol mechanisms use standard MAC command frames (direct and indirect communication), and all information for synchronisation and configuration of ARounD communication is included in the payload of IEEE 802.15.4 MAC command frames. For the sake of convenience, “ARounD frame” is used to refer to the synchronisation and configuration frames of the proposed approach.

#### 3.4.1. Synchronisation and Configuration of the Involved Nodes

**Figure 11:**
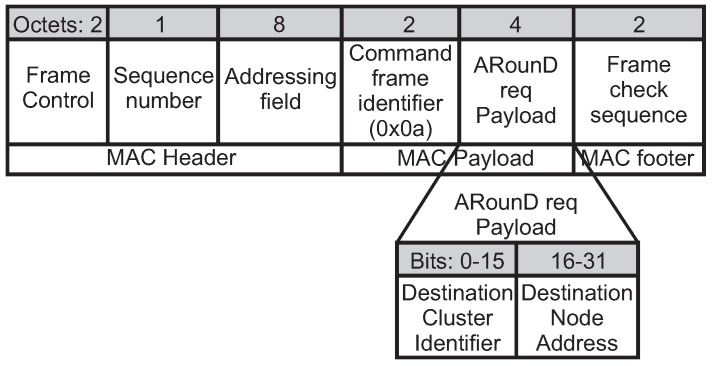
Format of the *ARounD-req* frame.

The setup of a new ARounD communication path is triggered by the source node using an *ARounD request command* frame (ARounD req). The *ARounD req* frame is transmitted during the CAP period towards the PAN coordinator. Figure 11 illustrates the format of this frame. The MAC header fields contain the frame control, sequence number and addressing field. The *Addressing field* contains the *destination Cluster Identifier* and *destination address* fields, which corresponds to PAN coordinator. In turn, the *source Cluster Identifier* and *source address* fields correspond to the source node that is requesting the ARounD communication path. The 16-bit short address is used as addressing type. In order to identify the *ARounD req* frame, the value of the *command frame identifier* field is defined as *0x0a*, which is a value not used by IEEE 802.15.4. The *ARounD req payload* identifies the cluster and address of the destination node.

**Figure 12:**
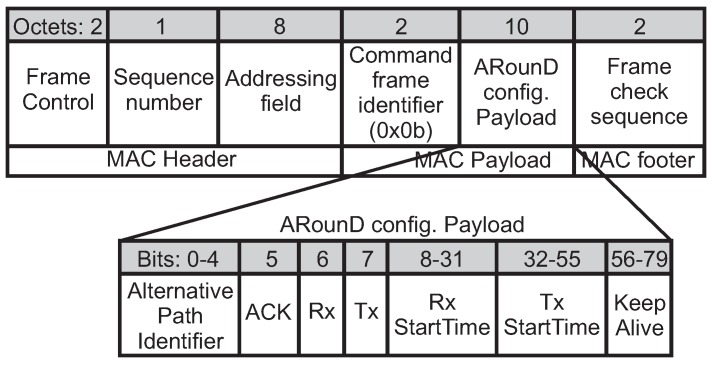
Format of the *ARounD-conf* frame.

Within this context, after receiving an *ARounD req* frame and performing the ARounD algorithms, the PAN coordinator uses an *ARounD configuration command* frame (ARounD conf) to establish/maintain/close an ARounD communication path. Figure 12 illustrates the format of this frame. The *Addressing field* contains the *destination Cluster Identifier* and *destination address* fields, which correspond to the RP node to be configured. In turn, the *source Cluster Identifier* and *source address* fields correspond to the PAN coordinator. In order to identify the *ARounD conf* frame, the value of the *command frame identifier* field is defined as *0x0b*, which is a value not used by IEEE 802.15.4.

The *ARounD configuration payload* contains the configuration fields of the RP node specified in the addressing field. The *Alternative path identifier* field defines the alternative path to be configured, which allows to set up to 32 message streams at the same time. The *ACK* field is set to 1 if the ARounD communication supports acknowledgements; otherwise, this field is set to 0. The *Tx* and *Rx* fields are set to 1 if the involved node is a receiver or/and transmitter, respectively; otherwise, these fields should be set to 0. Note that, if the *Rx* field is set to 1, the *Rx StartTime* field should be configured to the time (*MS_Offset*) when the node will wake up in receiver mode. In turn, if the *Tx* field is set to 1, the *Tx StartTime* field should be configured to the time (*MS_Offset*) when the node will wake up to send data (transmitter mode). In addition, the *Keep alive* field specifies the period (seconds) during which the alternative path should be kept active. The PAN coordinator will send the *ARounD conf* frames along the cluster coordinators until reaching all the involved nodes, using indirect communication mechanisms.

**Figure 13:**
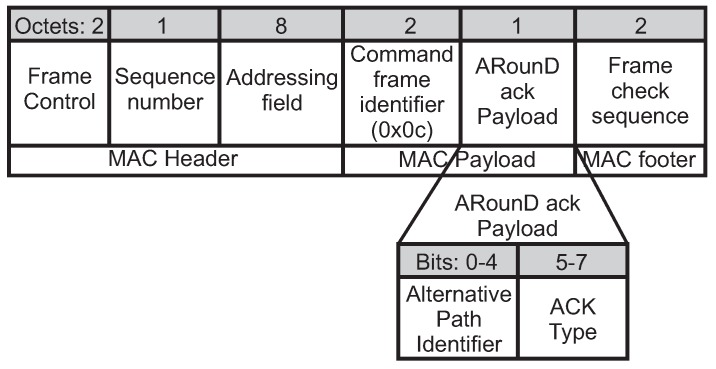
Format of the *ARounD acknowledgement command* frame.

After receiving the *ARounD conf* frame, the involved node sends an acknowledgement frame to inform the PAN coordinator, using the *ARounD ack command* frame. Figure 13 illustrates the format of this frame. In order to identify the *ARounD ack* frame, it is used the value *0x0c* in the *command frame identifier* field, which is a value not used by the IEEE 802.15.4 standard. The *Addressing field*, *destination Cluster Identifier* and *destination address* fields correspond to the PAN coordinator. In turn, the *source Cluster Identifier* and *source address* fields correspond to the configured node. The 16-bit short address is used for addressing purposes.

The *ARounD ACK payload* field is composed of the *alternative path identify* field (5-bits) to identify the alternative path and the *ACK type* field to specify the *acknowledgement frame type*. Table 1 summarises the *ACK frame* types used in the ARounD communication scheme.

After receiving the acknowledgements from the involved nodes (source, destination and RP nodes), the PAN coordinator will authorise the start of the new ARounD message stream using an *ARounD authorisation command* frame (ARounD auth). Whenever the PAN coordinator does not receive all the required acknowledgements, it will retry to configure the missing nodes for a number of attempts defined at the application level. This parameter is configured based on timing requirements imposed by the supported application. If the configuration does not succeed after the number of defined attempts, the ARounD path will not be created. Note that involved nodes just start the ARounD communication after the authorisation of PAN Coordinator, avoiding the waste of energy.

**Figure 14:**
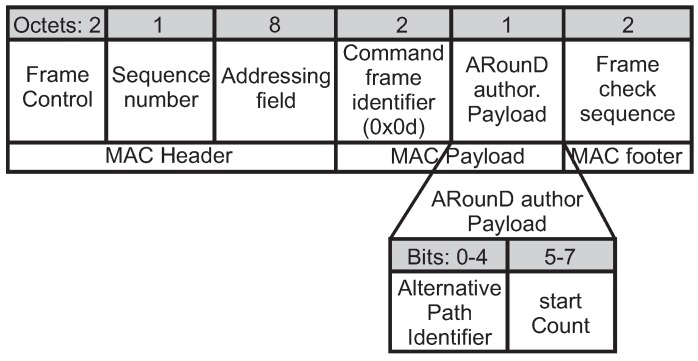
Format of the *ARounD auth* frame.

The *ARounD auth* frame is sent to the involved nodes using indirect communication mechanisms. Figure 14 illustrates the format of this frame. The value *0x0d* is used for the *command frame identifier* field, which is a value also not used by the standard. Similarly to the configuration procedure, the *Addressing field* contains the *destination Cluster Identifier* and *destination address* fields, which corresponds to the node to be authorised. In turn, the *source Cluster Identifier* and *source address* fields correspond to the PAN coordinator.

#### 3.4.2. Closing the ARounD Communication

As presented in the previous subsection, the ARounD communication is configured to work during a given time, which is specified in the *keep alive* field. Nevertheless, the ARounD communication also provides an explicit mechanism to close an on-going alternative path.

**Figure 15:**
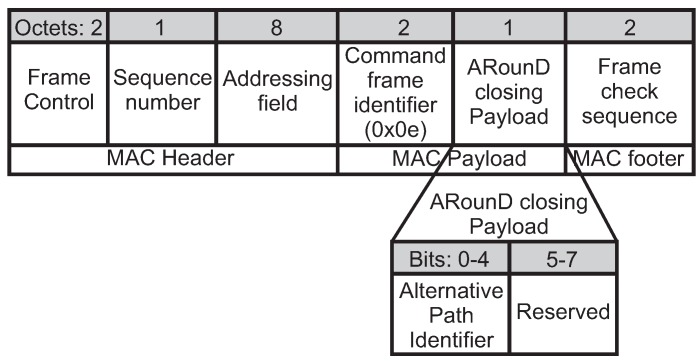
Format of the *ARounD closing command* frame.

The *ARounD communication closing* mechanism is launched by the PAN coordinator, which sends an *ARounD closing* frame to all the involved nodes. Figure 15 illustrates the format of this frame. After receiving this frame, the involved nodes immediately finish the ARounD communication. Finally, each involved node sends to PAN coordinator an *ARounD ack* frame (ACK type field set to 001).

**Figure 16:**
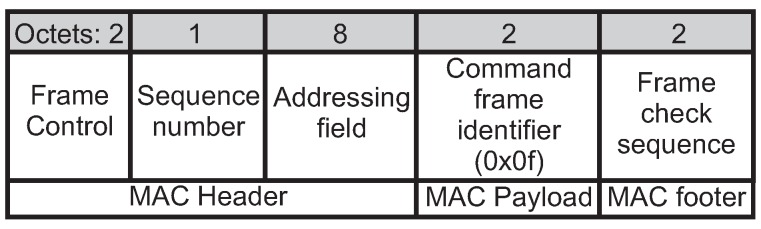
Format of the *ARounD hello command* frame.

The ARounD communication also enables the PAN coordinator to verify if all the involved nodes are still alive, using an *ARounD hello* frame. Figure 16 illustrates the format of this frame. Note that the MAC payload is composed of the *command frame identifier* field only (with value set to *0x0f*, which is not used by the IEEE 802.15.4), in order to identify this frame. The PAN coordinator may query a specific node to confirm if it is still alive. This node replies with an *ARounD ack* frame (*ACK type* field set to 010).

## 4. Simulation Assessment of the ARounD Communication Scheme

In the previous sections, a set of communication mechanisms (ARounD) were proposed, enabling the setting-up of peer-to-peer communication paths upon a cluster-tree wireless sensor network.

In this section, we present a simulation assessment of the proposed communication scheme. The rationale behind this assessment is to compare the behaviour of a source-to-destination message stream when its messages are transferred using the ARounD communication scheme vs. using the standard cluster-tree routing scheme (IEEE 802.15.4/ZigBee parent-child relationships). The cluster-tree network is shared with a set of background message streams, that are sending convergecast traffic towards the PAN coordinator.

For this simulation assessment, it was used the Castalia Simulator [63]. Castalia (The Castalia Simulator for Wireless Sensor Network: https://castalia.forge.nicta.com.au.) is an open-source discrete event simulator for wireless sensor networks (WSN), Body Area Networks (BAN) and general low-power embedded networks. It was developed at National ICT Australia (NICTA) and is based on the OMNeT++ platform widely used by researchers and developers to test communication protocols using realistic wireless channel and radio models. According to [64], Castalia has become a very popular simulator as it was developed for research purposes. Castalia implements an advanced wireless channel model based on empirically measured data. Also, the simulator provides radio models based on real low-power communication radios. Moreover, important features to simulate WSNs are available, such as: realistic node behaviour, node *clock drift*, and energy consumption models.

Castalia provides an IEEE 802.15.4 model. However, this model is quite limited. Basically, it only implements the CSMA-CA functionality and a beacon-enabled star topology, including an association procedure, direct data transfer mode, and GTS communication. Therefore, we developed the CT-SIM simulation model [65], which includes a series of functionalities, such as: cluster-tree formation procedure, network scheduling, hierarchical addressing scheme, direct and indirect data communication, collision domain definition, data communication to the sink node (PAN coordinator), and source-to-destination message streams. The ARounD communication mechanisms were implemented upon the CT-SIM simulation model.

### 4.1. Simulation Environment

In order to assess the ARounD communication scheme, it was defined a simulation environment with size 200 m × 200 m, containing 503 sensor nodes (one PAN coordinator, one pair of source and destination nodes, plus 500 general purpose nodes). This assessment adopted the radio model CC2420 (Texas Instruments/Chipcon CC2420 Datasheet: http://www.ti.com/product/CC2420/technicaldocuments), which is compliant with IEEE 802.15.4. Moreover, a linear energy model provided by Castalia was used for simulations. The initial energy for all nodes was set to 18,720 Joules, which is the typical energy for two AA batteries. As radio propagation model, we adopted the *unit disc* model, where the range of the disk was defined to 55 m. In addition, it was used an interference model, where concurrent transmissions generate collisions at the receiver.

For the nodes’ deployment, node 0 was set as the PAN coordinator and nodes 1 and 2 as the source and destination nodes for the source-to-destination message stream, respectively. These nodes are statically deployed in the communication environment. The PAN coordinator is located in the central position of the field (100 m, 100 m). For nodes 1 and 2, two different scenarios were defined, in order to avoid biased assessments. Firstly, nodes 1 and 2 were deployed in positions (30 m, 30 m) and (170 m, 30 m), respectively, in adjacent quadrants (*Scenario 1*). This configuration was used to have a balanced deployment. Secondly, nodes 1 and 2 were deployed in positions (30 m, 30 m) and (170 m, 170 m), respectively, in opposite quadrants (Scenario 2). This configuration was used to configure a communication scenario, where the PAN coordinator is located between the source and destination nodes, forcing the peer-to-peer communication through nodes deployed in more congested areas of the network.

**Figure 17:**
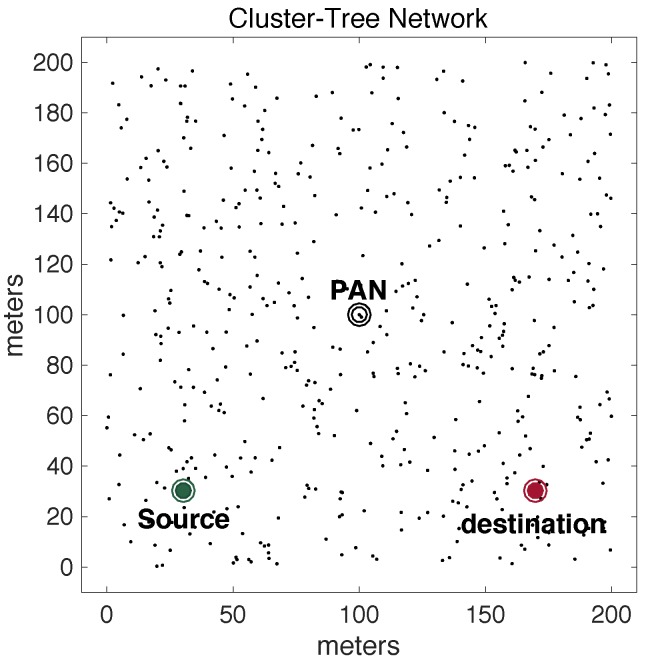
Scenario 1: nodes 1 and 2 in adjacent quadrants.

**Figure 18:**
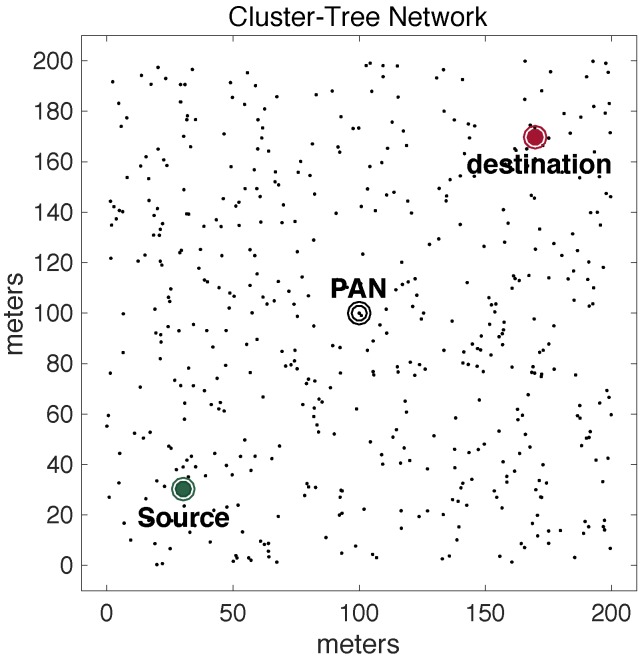
Scenario 2: nodes 1 and 2 in opposite quadrants.

The other 500 general-purpose nodes were randomly deployed in the environment. Basically, five different physical topologies were generated for setting-up the position of these nodes, which were used for the assessment of the ARounD communication mechanisms. Thus, we have 10 different deployments: five topologies for *Scenario 1* and five topologies for *Scenario 2*. For each topology, 10 simulations were run with different sets of random variables. The results presented along this section correspond to the average of results from these set of simulations. Figure 17 illustrates one of 5 physical topologies considered for *Scenario 1*, while Figure 18 illustrates one of 5 physical topologies considered for *Scenario 2*.

The cluster-tree formation is based on the IEEE 802.15.4/ZigBee standard. The PAN coordinator performs the cluster-tree scheduling and checks if the current cluster-tree configuration is schedulable. Table 2 summarises the most important configuration parameters used in the simulations and pointed out in this section.

#### 4.1.1. Characterisation of Data Traffic

After finishing the cluster-tree network formation, sensor nodes start the *data communication phase*. In the simulations, two data communication patterns were defined, as follows:

- *background data traffic*: 500 general-purpose sensor nodes sending convergecast periodic data towards the sink node (PAN coordinator).
- *source-to-destination data traffic*: a specific source node sending periodic data to a specific destination node.

In the simulations, both the PAN coordinator and the source and destination nodes do not generate any background data traffic. Thus, there are 500 sensor nodes generating background data traffic. Each sensor node generates 1000 data frames. Considering this setup, the background traffic load corresponds to a sequence of 500,000 data frames sent by 500 randomly deployed sensor nodes to the PAN coordinator (sink node). Importantly, the cluster-heads do not perform any data aggregation or data fusion operation [66], which implies that all background traffic is forwarded towards the sink node. In future assessments, these techniques will be also implemented in the *ARounD simulation model* in order to assess the behaviour of different data aggregation/fusion techniques in distributed wide-scale applications.

**Figure 19:**
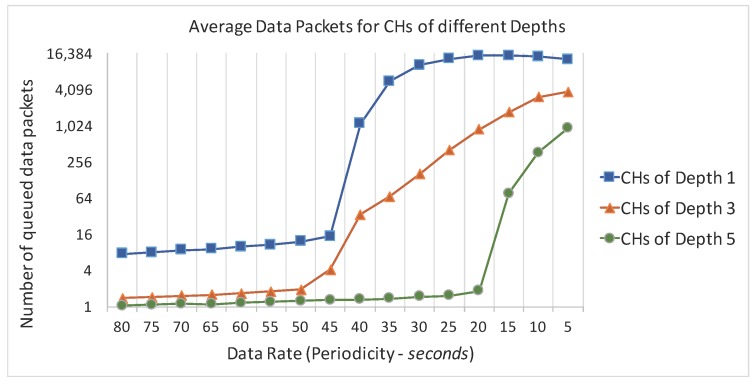
Average number of queued data packets in MAC buffers.

In order to select an adequate value for the background data rate, we performed a set of experiments to evaluate the average number of queued packets at the cluster-head nodes, using different background data rates. The intention was to select an active but not congested communication environment to perform the simulation assessment of the ARounD communication mechanisms. Figure 19 illustrates the average number of packets in the MAC buffers of the cluster-heads at different depths of the cluster-tree network (the PAN coordinator corresponds to depth 0).

Note that, from data rates above 1 packet generated every 45 s by each sensor node, the number of queued packets exponentially increases, meaning that these cluster-heads will be quickly facing queue overflows. For a data rate of 1 packet every 15 s, the scenario is much worse, as it will result in a fully congested environment, where a huge number of data packets are automatically dropped by the network.

According to this network behaviour, we considered two different perspectives for setting-up the background data traffic: application load or network load perspectives. Regarding the application load perspective, the data rates for the background traffic were defined from a periodicity of 60 s (1 generated packet every 60 s) up to a periodicity of 40 s (1 generated packet every 40 s). These values may correspond to the data rates of typical monitoring applications. On the other hand, regarding the network load perspective, the number of background messages generated during one beacon interval ranges from 0 to 50 messages per beacon interval. Therefore, the results in this simulation assessment are presented both in application load and in network load perspectives.

The source-to-destination data traffic is set for node 1, which sends 10,000 data frames to node 2 according to the following data rate configurations: 1 message every 4 s (periodicity of 4 s) and 1 message every 8 s (periodicity of 8 s). These are typical values that can be found for the transmission of video streams in wireless visual sensor networks [67]. This peer-to-peer message stream is started after a simulation time of 100 s.

#### 4.1.2. Performance Metrics

The aim of this simulation assessment is to evaluate the behaviour of the ARounD communication scheme when compared to the use of cluster-tree routing and its impact over the background communication traffic. For the sake of convenience, we adopted the name *Standard Approach* for the cluster-tree routing and *ARounD Approach* for the ARounD communication routing. The following performance metrics were used for the assessment of the network behaviour:

- *End-to-end Delay*: time interval between the data frame generation at the application layer of the source node and its reception at the application layer of the destination node.
- *Packet Loss Rate*: the percentage of packets lost during the communication, considering the number of data packets successfully received at the destination node and the number of packets generated by the source node.

### 4.2. Results and Discussion

Table 3 illustrates the results obtained from the network formation process (*Scenarios 1* and *2*), concerning the average number of generated clusters during the cluster-tree formation, the average maximum depth of the cluster-tree network and the average number of children per cluster. One important result is that all the communication environment was fully covered, meaning that all nodes were associated with a particular cluster-head (no orphan nodes).

Regarding the source-to-destination communication, Table 4 presents some relevant information about nodes 1 (source) and 2 (destination), and the source-to-destination message stream.

**Figure 20:**
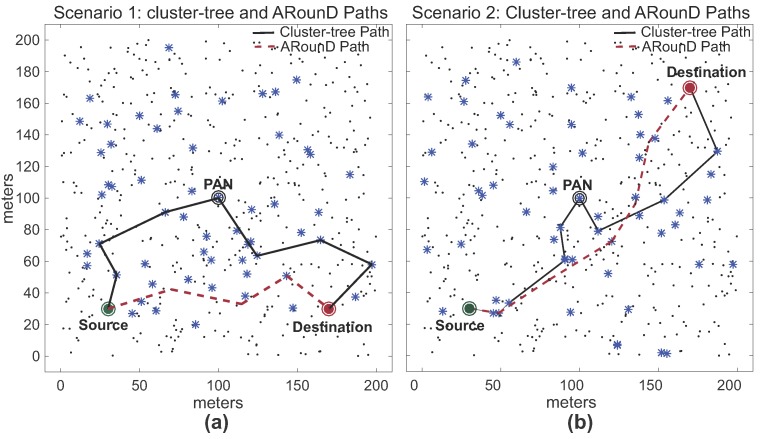
Cluster-tree and ARounD paths for one of physical topologies: (**a**) Scenario 1; (**b**) Scenario 2.

Note that, as expected for *Scenario 1*, the ARounD path is significantly shorter than the cluster-tree path. This is basically due to the selection of the shortest inter-cluster path between the source and destination nodes. For *Scenario 2*, the difference between the cluster-tree and ARounD paths is smaller, as the PAN coordinator is located between source and destination nodes. However, even though cluster-tree and ARounD paths are equivalent in the number of hops, the main advantage of ARounD is that it prioritises leaf nodes to build the routing path, allowing to save energy of cluster-tree nodes. Figure 20 illustrates one of the physical topologies for *Scenarios 1* and *2*. Note that both ARounD paths are composed mostly of leaf nodes.

Although the ARounD paths are usually smaller than cluster-tree paths, the main target of these simulations is to evaluate the performance of the message stream using the ARounD scheme compared to the IEEE 802.15.4/ZigBee standard cluster-tree routing, regarding end-to-end delays and successfully received data messages.

**Figure 21:**
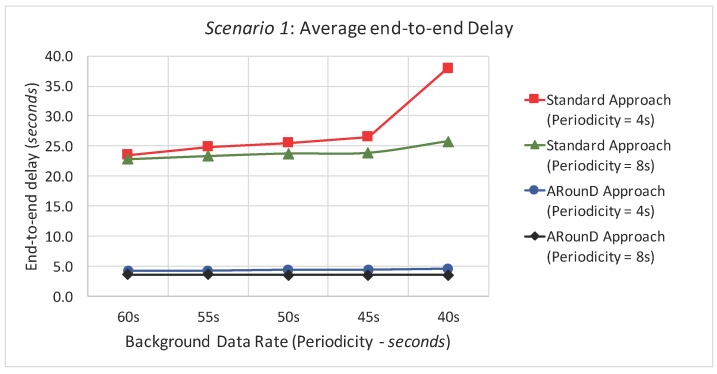
End-to-end Delay for ARounD Approach vs. Standard Approach considering Scenario 1, with different data rates for the background traffic.

Within this context, and according to the application perspective, Figure 21 shows the average end-to-end delay of the source-to-destination message stream for simulation *Scenario 1* (five physical topologies, under different background and source-to-destination data rates). The ARounD approach provides a smaller end-to-end delay compared to the IEEE 802.15.4 standard tree routing (Standard approach) for all background and source-to-destination data rates. This is due not just to the shorter inter-cluster path between the source and destination nodes; the main reason is that an ARounD path is scheduled within just one beacon interval, whereas a standard path is scheduled over several beacon intervals. This is one of the major disadvantages of cluster-tree path scheduling, where either a top-down or a bottom-up approach is used for scheduling the traffic (but not both). It is well-known that bottom-up scheduling approaches favour upstream traffic (from sensors to the PAN coordinator), while top-down approaches favour downstream traffic [39]. Moreover, the contention and transmission attempts of the CSMA-CA algorithm also impact the end-to-end communication delay in more congested areas of the wide-scale cluster-tree (near the PAN coordinator) [19].

It can be also observed that, as the background data rate increases, the average end-to-end delay for the standard cluster-tree approach also increases. This is mainly due to the accumulation of queued data packets in the cluster-heads near the PAN coordinator [19], causing further delays to the newly arriving packets. However, as the ARounD scheme defines an alternative path using RP nodes (which are mainly leaf nodes), the end-to-end delay for the source-to-destination traffic is just slightly affected. As it was expectable, the average end-to-end delay is highly dependent on the background traffic load for the standard cluster-tree approach, as the source-to-destination traffic is sharing the same MAC queues with the background traffic. On the other hand, in the ARounD scheme, as the source-to-destination traffic almost does not share RP nodes with the background traffic, the average end-to-end delay keeps constant for the different data rates.

**Figure 22:**
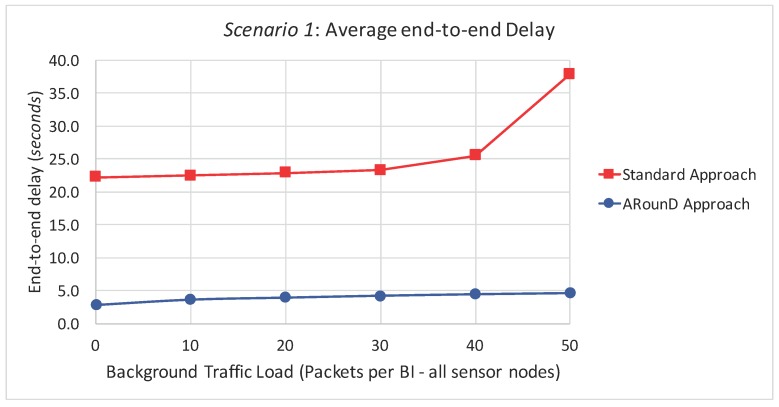
End-to-end Delay for ARounD Approach vs. Standard Approach in Scenario 1, considering different background traffic loads.

Figure 22 illustrates the average end-to-end delays of the source-to-destination message stream according to the network load perspective (as a function of the traffic load imposed by sensor nodes). For this set of simulations, we just considered a data rate of 1 packet every 4 s. The rationale of this set of simulations is to evaluate the behaviour of source-to-destination traffic delays under different traffic loads imposed by sensor nodes (including a scenario with no background traffic). As it can be observed, the ARounD approach provides smaller and bounded end-to-end delays for the source-to-destination traffic, even under different traffic loads. On the other hand, the standard tree routing approach tends to increase as the background traffic increases. Note also that, even without background traffic (no interference), the end-to-end delays for the source-to-destination traffic is higher, when using the standard tree routing, due to the higher number of beacon intervals that are required to transmit data frames.

**Figure 23:**
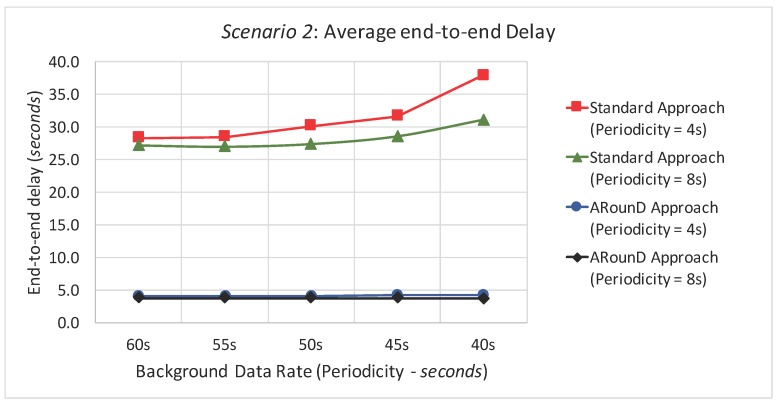
End-to-end Delay for ARounD Approach vs. Standard Approach in Scenario 2, considering different data rates for the background traffic.

**Figure 24:**
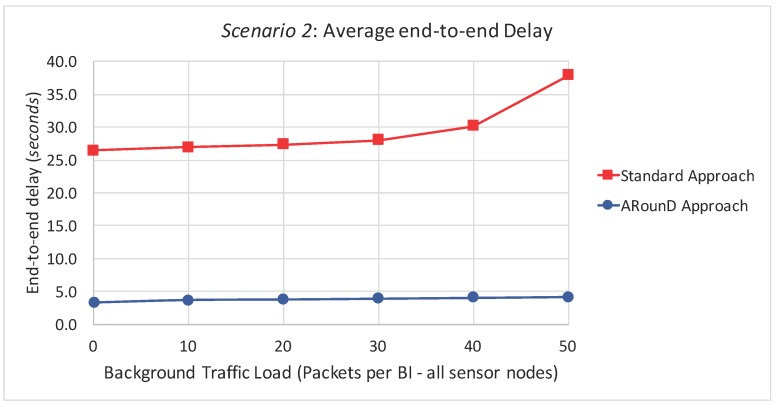
End-to-end Delay for ARounD Approach vs. Standard Approach considering Scenario 2, considering different traffic loads imposed by sensor nodes.

Figure 23 and Figure 24 illustrate the average end-to-end delay of the source-to-destination message stream for simulation *Scenario 2* (five physical topologies, under different background and source-to-destination data rates), for both the application and network load perspectives. Also, the ARounD approach provides a smaller end-to-end delay when compared to the Standard approach for all background and source-to-destination data rates and traffic loads.

**Figure 25:**
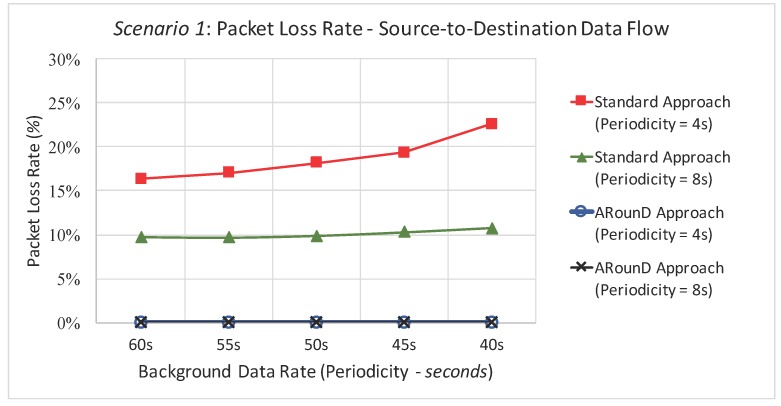
Packet Loss Rate for ARounD Approach vs. Standard Approach for Scenario 1, considering different data rates for the background traffic.

**Figure 26:**
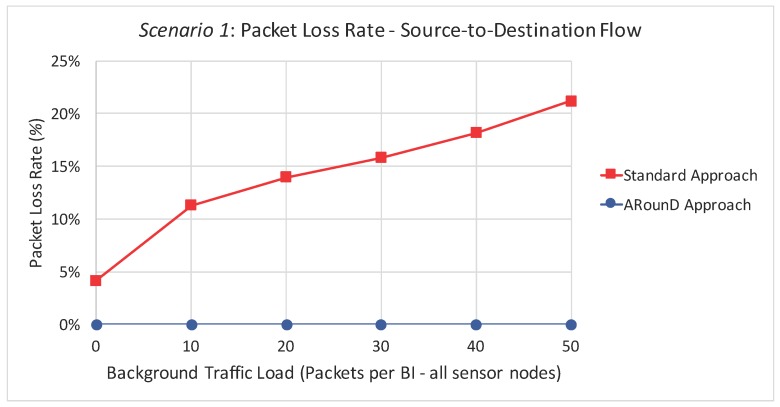
Packet Loss Rate for ARounD Approach vs. Standard Approach for Scenario 1, considering different traffic loads imposed by sensor nodes.

Figure 25 illustrates the packet loss rate due to buffer overflows as a function of the background traffic data rates for Scenario 1, while Figure 26 illustrates same metric as a function of the traffic load imposed by all sensor nodes. These results highlight that the ARounD scheme successfully delivers all the source-to-destination messages, while the standard cluster-tree path is prone to a high number of packet losses due to network congestion issues. The main reason is that, as the ARounD communication scheme operates during the inactivity period of the clusters, it does not need to contend for the channel access, which avoids packet losses caused by collisions. Additionally, as ARounD prioritises the use of leaf nodes to convey its source-to-destination packets, it avoids the use of more congested CH nodes and therefore the number of packet discards due to network congestion is negligible. Finally, note also that, although the availability of GTS mechanism to transmit time-sensitive packets without contention, the main weakness of this mechanism is its limited number of available slots [68].

**Figure 27:**
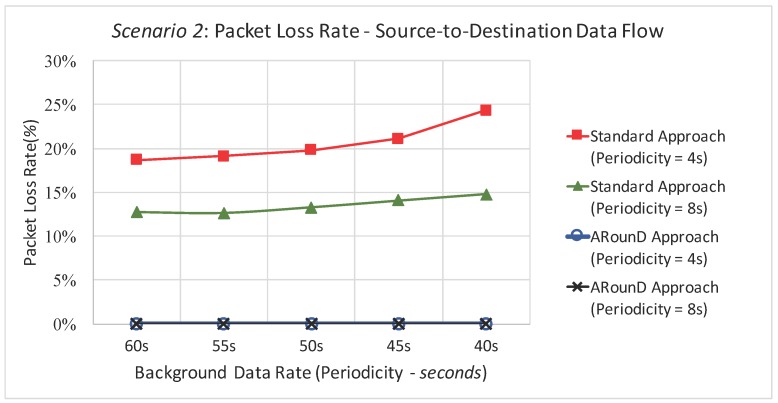
Packet Loss Rate for ARounD Approach vs. Standard Approach for Scenario 2, considering different data rates for the background traffic.

**Figure 28:**
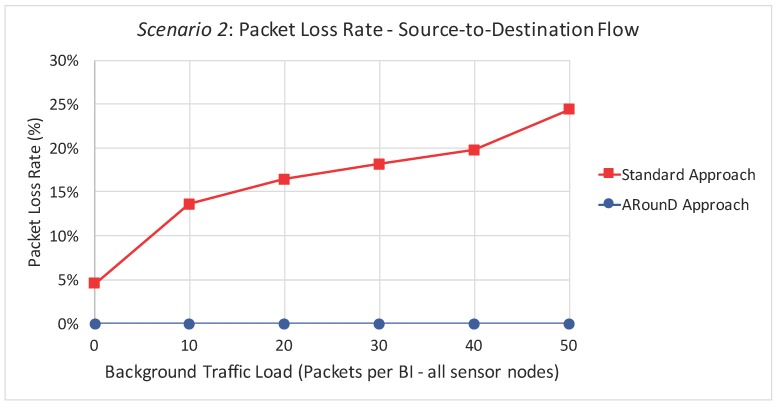
Packet Loss Rate for ARounD Approach vs. Standard Approach for Scenario 2, considering different traffic loads imposed by sensor nodes.

Following the same rationale, Figure 27 illustrates the packet loss rate as function of the background traffic data rates, while Figure 28 illustrates the packet loss rate as function of the network loads imposed by sensor nodes for the *Scenario 2*. Note that a similar behaviour was observed for this scenario, which shows that the ARounD approach can provide a better performance regarding to packet loss rate, when compared to the Standard Approach.

**Figure 29:**
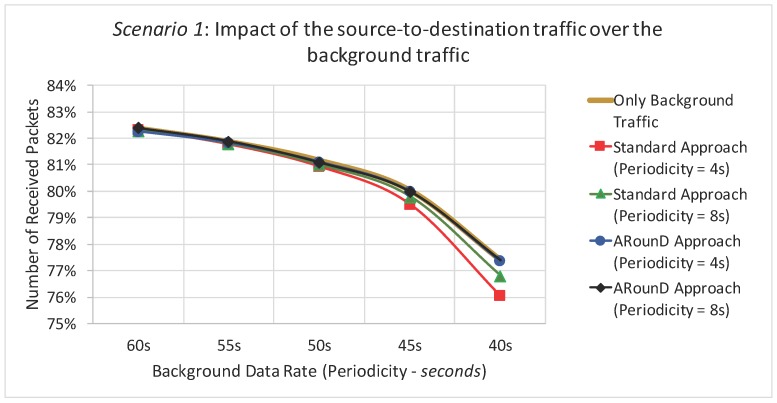
Impact of the source-to-destination traffic over the background traffic (Scenario 1).

An important issue is also the assessment of the impact of the source-to-destination traffic over the background traffic, when using the ARounD approach. For this purpose, a set of simulations were performed considering only background data traffic, in order to capture its behaviour without the source-to-destination traffic versus its behaviour considering also the source-to-destination data traffic (for different background data rates – application point of view). Figure 29 illustrates the number of received packets for *Scenario 1*, where it is clear that Standard approach has a negligible impact, but greater than those caused by the ARounD communication scheme. In fact, as the standard approach also uses the cluster-tree path to transfer source-to-destination traffic, the cluster-tree network has more packets contending for the communication path, increasing the packet loss rate. A similar behaviour was also observed for *Scenario 2* and when varying the traffic load (network load perspective).

**Figure 30:**
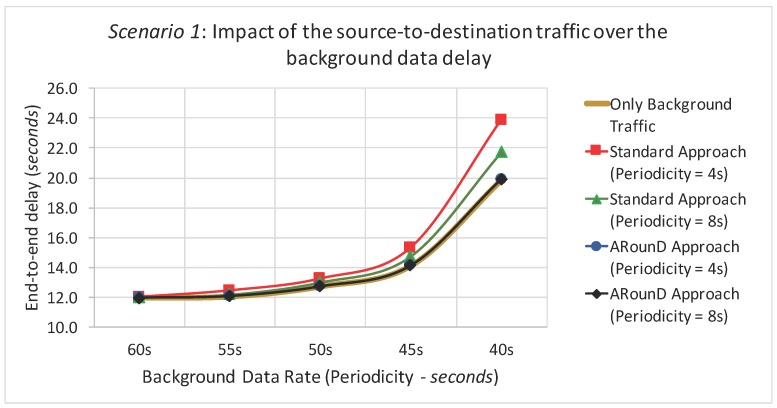
Impact of the source-to-destination data traffic (ARounD and Cluster-tree approaches) over the background data traffic delay, considering the Scenario 1.

It was also assessed the impact of source-to-destination data traffic upon the average end-to-end delay of background data traffic. As it can be seen in Figure 30, the ARounD communication traffic does not interfere with the background traffic. The main reason is that ARounD defines an alternative path to transmit source-to-destination traffic. Conversely, as the standard approach uses the cluster-tree path to transmit its source-to-destination data traffic, the number of packets in the MAC buffers tends to increase, impacting the background traffic end-to-end delay. A similar behaviour was also observed for *Scenario 2*.

**Figure 31:**
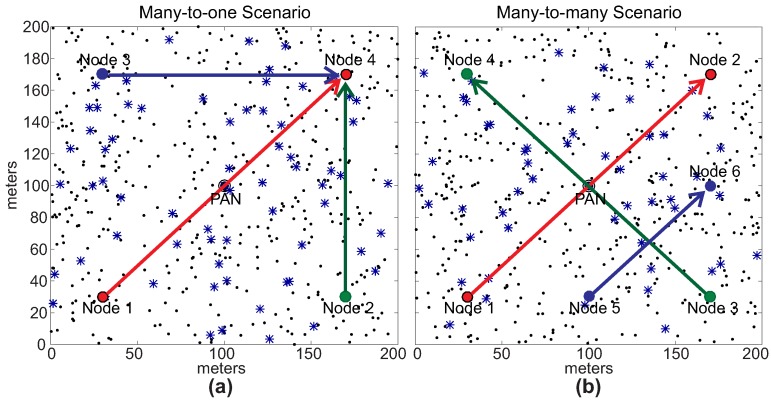
Scenarios considering multiples source-to-destination traffics: (**a**) Many-to-one message streams; (**b**) Many-to-many message streams.

Finally, a set of experiments using multiples message streams were also assessed. The rationale behind these experiments is to show the capability of the proposed communication scheme to support multiple source-to-destination communication message streams. More specifically, we considered two communication scenarios with different traffic patterns for the source-to-destination traffic: many-to-one (three sources to a unique destination) and many-to-many (three sources and three destinations). Figure 31a illustrates a many-to-one communication scenario, where three sources (nodes 1, 2 and 3) send messages to a unique destination (node 4), while Figure 31b illustrates a many-to-many communication scenario, where three sources (nodes 1, 3 and 5) send messages to different destinations (nodes 2, 4 and 6), respectively.

**Figure 32:**
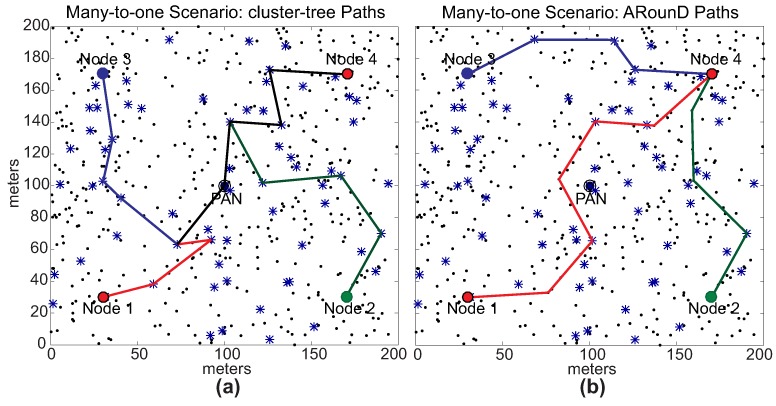
One of physical topologies for many-to-one scenario: (**a**) Cluster-tree paths of message streams; (**b**) ARounD paths of message streams.

Figure 32 illustrates one of the physical topologies for the many-to-one scenario. Figure 32a illustrates the cluster-tree paths, where all communication paths follow the tree routing towards the PAN coordinator, generating more congestion for coordinator nodes. In turn, Figure 32b shows the ARounD paths, where the communication paths are performed by repeater nodes, avoiding the cluster-tree path.

**Figure 33:**
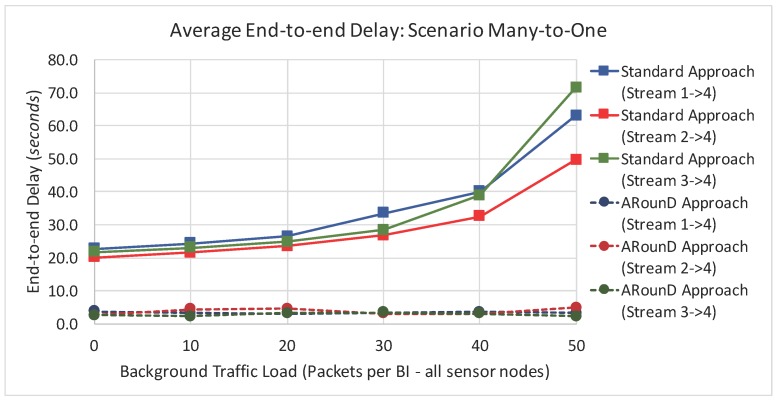
Average end-to-end delay for multiple many-to-one message streams, considering the ARounD Approach vs. Standard Approach under different traffic loads imposed by sensor nodes.

Concerning the communication behaviour, Figure 33 shows the average end-to-end communication delays for many-to-one source-to-destination message streams, as a function of the background traffic load imposed by sensor nodes. As it can be observed, the ARounD approach provides smaller and constant end-to-end delays for all message streams, even for different background traffic loads. As previously mentioned, ARounD schedules message streams during the inactive periods of the sensor nodes, which allows communication without contention for the channel access and avoids interference with the cluster-tree network. On the other hand, the standard approach provides higher end-to-end communication delays for multiples message streams, that tend to increase as the background traffic increases.

**Figure 34:**
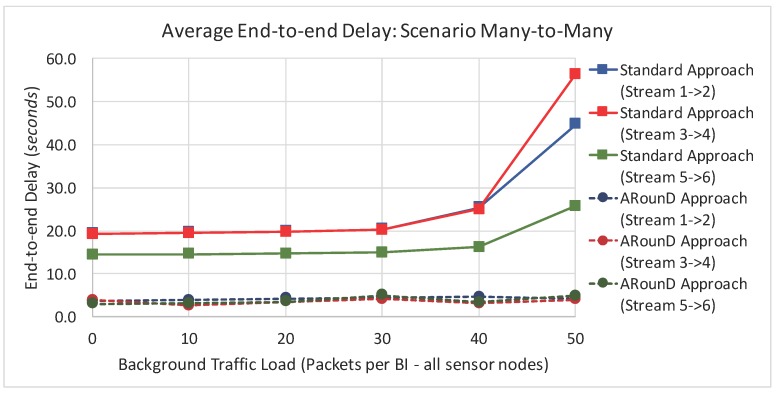
Average end-to-end delay for multiple many-to-many message streams, considering the ARounD Approach vs. Standard Approach under different traffic loads imposed by sensor nodes.

Figure 34 illustrates a similar behaviour for the average end-to-end communication delays for many-to-many source-to-destination message streams.

**Figure 35:**
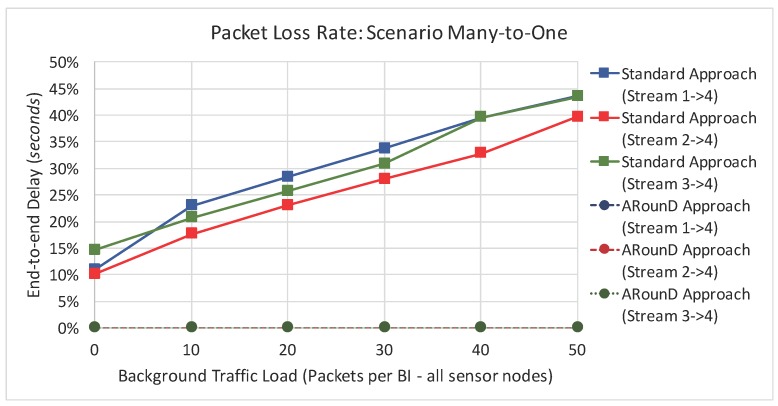
Packet Loss Rate for multiple many-to-one message streams, considering the ARounD Approach vs. Standard Approach under different traffic loads imposed by sensor nodes.

**Figure 36:**
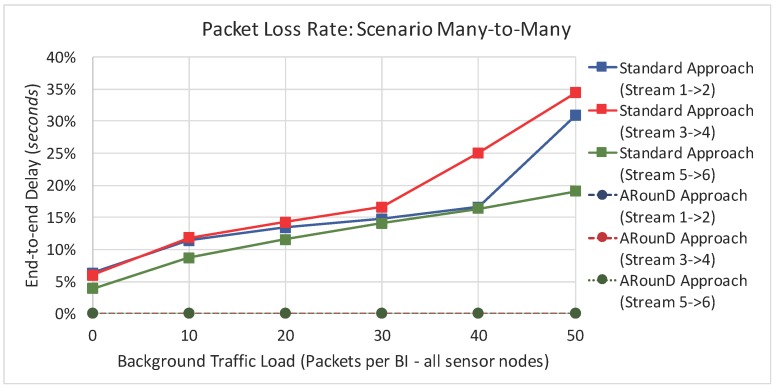
Packet Loss Rate for multiple many-to-many message streams, considering the ARounD Approach vs. Standard Approach under different traffic loads imposed by sensor nodes.

Finally, Figure 35 and Figure 36 illustrate the packet loss rates for both many-to-one and many-to-many scenarios, respectively. The results are also presented as a function of the background traffic load imposed by sensor nodes. Note that, the Standard approach presents higher packet loss rates due to collisions and discarded messages, while ARounD scheme successfully delivered all source-to-destination messages. As previously mentioned, ARounD approach avoids collisions and discarded messages by using alternative paths from the source node to destination node to forward messages without contending for the channel access. Note also that, as the background traffic load increases, the number of lost messages by using the Standard approach also increases.

## 5. Conclusions

In this paper, we present the ARounD (*Alternative-Route Definition*) communication scheme, that enables the definition of alternative communication paths for peer-to-peer message streams in IEEE 802.15.4/ZigBee-based cluster-tree WSNs. The ARounD communication scheme uses cluster-tree nodes during their inactive periods, in order avoid interferences with the cluster-tree network traffic. As a consequence, the ARounD communication scheme does not use the previously-defined cluster-tree paths, being able to improve several communication metrics such as: network congestion and end-to-end communication delays.

Simulation results highlight that the use of the ARounD communication scheme can significantly decrease the end-to-end communication delay for source-to-destination data traffic, because it defines shorter inter-cluster paths between source and destination nodes and also because it is able to schedule such alternative paths in just one beacon interval. Furthermore, as source-to-destination messages do not contend for the wireless channel access with other messages, it avoids additional delays and dropped packets due to collisions. It is also shown that the ARounD communication scheme has just a negligible impact upon the cluster-tree communication and can be used to support the transfer of messages, guaranteeing limited QoS requirements such as bandwidth and softly-bounded delays.

### Future Considerations

As future considerations, other metrics will also be used to define alternative paths, such as link quality and geographical location. With this, we intend to define improved ARounD paths, optimising the usage of communication network node resources. Besides, new functionalities will be added to the ARounD simulation model, such as: new scheduling approaches and aggregation and data fusion mechanisms, in order to prioritise specific communication traffics (upstream or downstream) and decrease the number of messages to be transferred in congested areas in the network. Also, we intend to implement the ARounD approach using a real hardware testbed.

## Figures and Tables

**Table 1:** ACK frame types for the ARounD communication scheme.

Frame Type Value	Description
000	ACK for an ARounD configuration frame
001	ACK for an ARounD closing frame
010	ACK for an ARounD hello frame
011–111	Reserved

**Table 2:** Simulation configuration.

Definition	Standard Value
Environment size	200 m × 200 m
Number of sensor nodes	503
Nodes sending Background Data	500
Radio model	Chipcon CC2420
Initial energy (per node)	18,720 J
Simulation time (each experiment)	85,000 s
Number of Background Data Frames (per node)	1000
Periodicity of background Data Rate	from 1 pkt every 60 s up to 1 pkt every 40 s
Number of Source-to-Destination Data Frames (Node 1)	10,000
Periodicity of Source-to-destination Data Rate	1 pkt every 4 s and 1 pkt every 8 s
physical Data Rate	250 kbps
*aBaseSlotDuration*	60
*aNumSuperframeSlots*	16
*aUnitBackoffPeriod*	20
*BeaconOrder*	9
*superframeOrder*	ranging from 0 to 4
*macMaxCSMABackoffs*	4
*macMaxFrameRetries*	2

**Table 3:** Information about the network formation.

Information	Value
Average number of clusters	59
Average maximum depth	5
Average number of children per cluster	8

**Table 4:** Information about source-to-destination message stream, considering the scenarios 1 and 2.

Information	Scenario 1	Scenario 2
Average depth of Node 1	3	4
Average depth of Node 2	3	4
Average number of hops of the standard paths	8	8
Average number of hops of the ARounD paths	4	7
